# Supplementary material for: Modeling the influences of non-local connectomic projections on geometrically constrained cortical dynamics
Source: PLoS Comput Biol. 2026 Aug 26;22(8):e1014673. doi: 10.1371/journal.pcbi.1014673 (PMC13552967; doi:10.1371/journal.pcbi.1014673)
Supplement: Supplementary file 1 [file pcbi.1014673.s001.pdf]

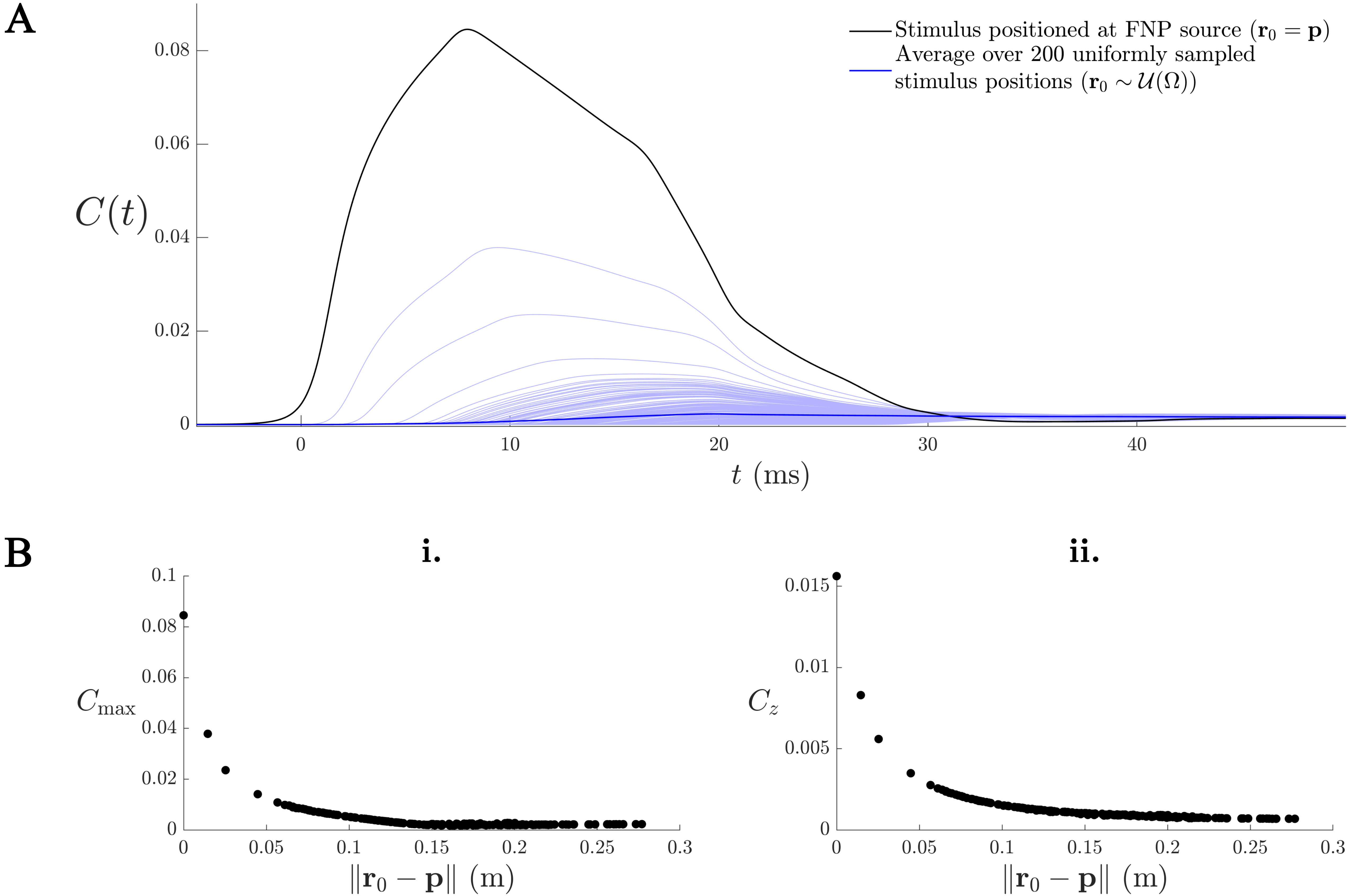

A
Stimulus positioned at FNP source ($\mathbf{r}_0 = \mathbf{p}$)
Average over 200 uniformly sampled stimulus positions ($\mathbf{r}_0 \sim \mathcal{U}(\Omega)$)
$C(t)$
0.08
0.06
0.04
0.02
0
0
10
20
30
40
t (ms)
B
i.
ii.
$C_{\max}$
0.1
0.08
0.06
0.04
0.02
0
0.05
0.1
0.15
0.2
0.25
0.3
$\|\mathbf{r}_0 - \mathbf{p}\|$ (m)
C_z
0.015
0.01
0.005
0
0.05
0.1
0.15
0.2
0.25
0.3
$\|\mathbf{r}_0 - \mathbf{p}\|$ (m)
