# Supplementary material for: Modeling the influences of non-local connectomic projections on geometrically constrained cortical dynamics
Source: PLoS Comput Biol. 2026 Aug 26;22(8):e1014673. doi: 10.1371/journal.pcbi.1014673 (PMC13552967; doi:10.1371/journal.pcbi.1014673)
Supplement: Supplementary file 2 [file pcbi.1014673.s002.pdf]

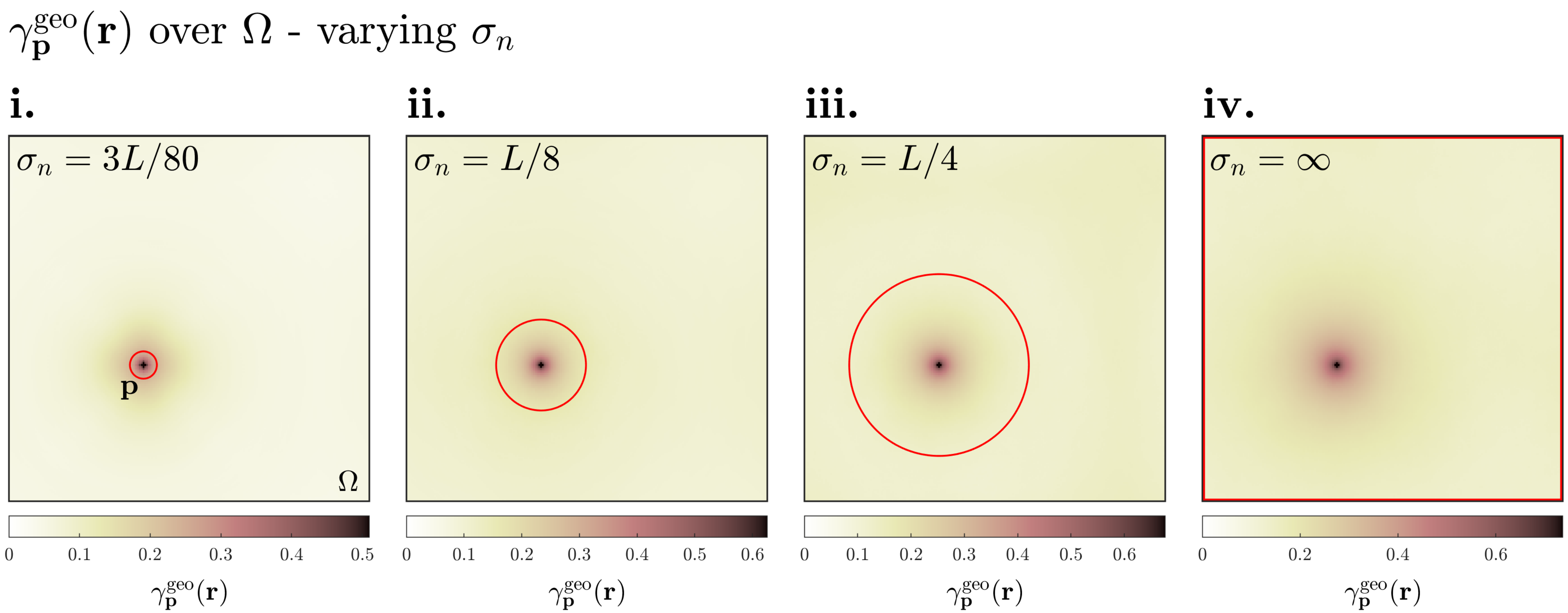

$\gamma_{\mathbf{p}}^{\text{geo}}(\mathbf{r})$ over Ω - varying σ_n
i.
ii.
iii.
iv.
$\sigma_n = 3L/80$
$\sigma_n = L/8$
$\sigma_n = L/4$
$\sigma_n = \infty$
$\mathbf{p}$
Ω
0 0.1 0.2 0.3 0.4 0.5
0 0.1 0.2 0.3 0.4 0.5 0.6
0 0.1 0.2 0.3 0.4 0.5 0.6
0 0.2 0.4 0.6
$\gamma_{\mathbf{p}}^{\text{geo}}(\mathbf{r})$
$\gamma_{\mathbf{p}}^{\text{geo}}(\mathbf{r})$
$\gamma_{\mathbf{p}}^{\text{geo}}(\mathbf{r})$
$\gamma_{\mathbf{p}}^{\text{geo}}(\mathbf{r})$
