# Supplementary material for: Modeling the influences of non-local connectomic projections on geometrically constrained cortical dynamics
Source: PLoS Comput Biol. 2026 Aug 26;22(8):e1014673. doi: 10.1371/journal.pcbi.1014673 (PMC13552967; doi:10.1371/journal.pcbi.1014673)
Supplement: Supplementary file 3 [file pcbi.1014673.s003.pdf]

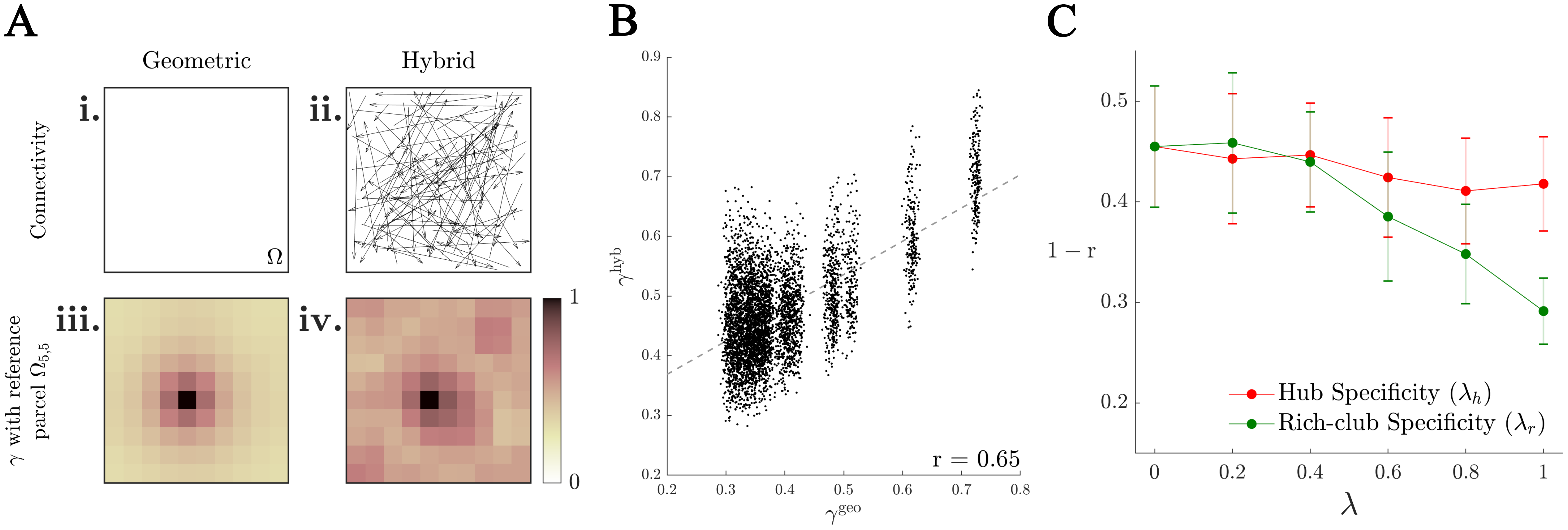
A
Geometric
Hybrid
Connectivity
i.
ii.
Ω
γ with reference parcel $\Omega_{5,5}$
iii.
iv.
1
0
B
γ^{hyb}
γ^{geo}
r = 0.65
0.2
0.3
0.4
0.5
0.6
0.7
0.8
0.9
C
1 − r
λ
0
0.2
0.4
0.6
0.8
1
0.5
0.4
0.3
0.2
Hub Specificity (λ_h)
Rich-club Specificity (λ_r)
