# Supplementary material for: Modeling the influences of non-local connectomic projections on geometrically constrained cortical dynamics
Source: PLoS Comput Biol. 2026 Aug 26;22(8):e1014673. doi: 10.1371/journal.pcbi.1014673 (PMC13552967; doi:10.1371/journal.pcbi.1014673)
Supplement: Supplementary file 4 [file pcbi.1014673.s004.pdf]

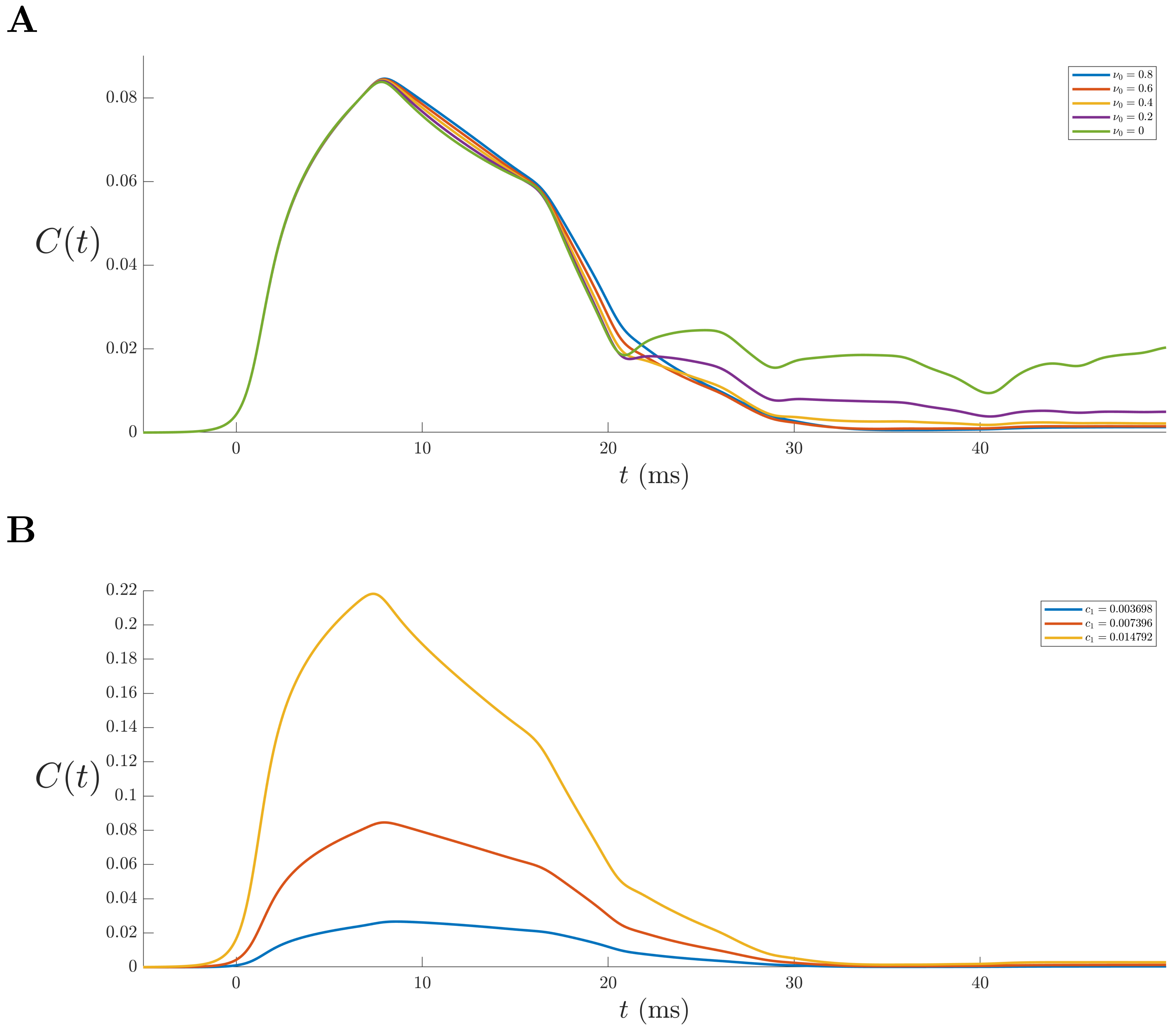
A
B
C(t)
t (ms)
$\nu_0 = 0.8$
$\nu_0 = 0.6$
$\nu_0 = 0.4$
$\nu_0 = 0.2$
$\nu_0 = 0$
$c_1 = 0.003698$
$c_1 = 0.007396$
$c_1 = 0.014792$
