# Supplementary material for: Modeling the influences of non-local connectomic projections on geometrically constrained cortical dynamics
Source: PLoS Comput Biol. 2026 Aug 26;22(8):e1014673. doi: 10.1371/journal.pcbi.1014673 (PMC13552967; doi:10.1371/journal.pcbi.1014673)
Supplement: Supplementary file 5 [file pcbi.1014673.s005.pdf]

# Supplementary Text — Modeling the influences of non-local connectomic projections on geometrically constrained cortical dynamics

## Section A: Model derivation

In this section, we derive the equation of the mathematical model introduced and used in this work, describing the relevant variables and physiological mechanisms throughout. The goal of this model is to approximate the spatiotemporal evolution of macroscale cortical activity—i.e., mean-field population-level activity on the cortical surface of a given cerebral hemisphere at millimeter lengthscales and above—from geometric propagation as traveling waves, concurrent with discrete connectomic propagation via fast-conducting non-local projections (FNPs) as rapid non-local interactions between specific populations on the surface. For simplicity, we derive the equation for the case of a single FNP, as this idealized case can be easily extended to multiple FNPs

The cortical surface (of either hemisphere) is mathematically represented as a closed continuous two-dimensional sheet denoted $\Omega$. At each point $\mathbf{r}$ on $\Omega$, we assign local geometric and non-local connectomic propagation to two separate populations. The locally propagating population, indexed $a = e$, has a spatially homogeneous and isotropic connectivity, and facilitates the geometric propagation. In other words, this population connects every pair of populations on $\Omega$ with connectivity that is completely dependent on the distance between the two points. The non-local population, indexed $a = w$, is an FNP that connects population $\mathbf{a}$ to population $\mathbf{b}$, and facilitates rapid propagation between this pair only. Finally, the inhibitory population, indexed $a = i$, only connects with other neurons in the same position. At a given point $\mathbf{r}$ over time $t$, we track two mean population-level activities for each population-type $a$: the mean firing rate of generated pulses (spikes) outgoing from the somas positioned at $\mathbf{r}$, $Q_a(\mathbf{r}, t)$, and the mean rate of pulses terminating at the synapses positioned at $\mathbf{r}$, $\phi_a(\mathbf{r}, t)$. We assume that $\phi_a(\mathbf{r}, 0) = Q_a(\mathbf{r}, 0) = 0$ for all $a$, since the temporal dynamics of all variables are driven by external input rather than initial conditions. The output variable of the model is $\phi_e(\mathbf{r}, t)$, per the construction of neural field models [1].

The propagator mechanisms of each population, which determine the influence of $Q_a$ on $\phi_a$ over space and time, is illustrated in Fig. A. Mathematically, the propagation of activity between two positions through the axons of population $a$ is represented by the Volterra integral equation

$$\phi_a(\mathbf{r}, t) = \int_\Omega d^2\mathbf{r}' \int_0^t dt'\; G_a(\mathbf{r}, t; \mathbf{r}', t')\, Q_a(\mathbf{r}', t') \text{ for all } (\mathbf{r}, t) \in \Omega \times [0, \infty)\,,\; a = e, i, w\,, \tag{S1}$$

where the propagator of type $a$, $G_a$, is the proportion of a pulse originating at a soma of type $a$ at position $\mathbf{r}'$ and time $t'$, that terminates at a synapse of type $a$ at position $\mathbf{r}$ and time $t$. Since all pulses terminate at a synapse, $G_a$ has unit integral over space and time. Population $e$ propagates activity between any two points on $\Omega$, in an accordance

to a distance-dependent connectivity rule. Given that $\Omega$ is two-dimensional, we use the connectivity rule used in the two-dimensional neural field developed by Robinson et al. [2]:

$$G_e(\mathbf{r},t;\mathbf{r}',t') = \frac{\gamma e^{-\gamma(t-t')}}{2\pi r}\frac{1}{\sqrt{r^2\gamma^2(t-t')^2 - |\mathbf{r}-\mathbf{r}'|^2}}\Theta(r\gamma(t-t') - |\mathbf{r}-\mathbf{r}'|)\,, \tag{S2}$$

where $r$ is the spatial lengthscale of the connectivity rule, and $\gamma$ is the axonal propagation speed divided by $r$. In contrast, population $w$ propagates activity strictly from point $\mathbf{a}$ to $\mathbf{b}$:

$$G_w(\mathbf{r},t;\mathbf{r}',t') = \delta(\mathbf{r}-\mathbf{b})\,\delta(\mathbf{r}'-\mathbf{a})\,\delta(t-t'-\tau), \tag{S3}$$

where $\tau$ is the conduction time for propagation in population $w$. Finally, population $i$ propagates activity only to neurons in the same position:

$$G_i(\mathbf{r},t;\mathbf{r}',t') = \delta(\mathbf{r}-\mathbf{r}')\,\delta(t-t'). \tag{S4}$$

Substituting the propagators of each type in Eqs (S2), (S3), (S4) to Eq. (S1) yields the equivalent equations:

$$\left(1 + \frac{2}{\gamma}\frac{\partial}{\partial t} + \frac{1}{\gamma^2}\frac{\partial^2}{\partial t^2} - r^2\nabla^2\right)\phi_e(\mathbf{r},t) = Q_e(\mathbf{r},t)\,; \tag{S5}$$

$$\phi_w(\mathbf{r},t) = \delta(\mathbf{r}-\mathbf{b})\,Q_w(\mathbf{a},t-\tau)\,; \tag{S6}$$

$$\phi_i(\mathbf{r},t) = Q_i(\mathbf{r},t)\,, \tag{S7}$$

where $\nabla^2$ is the Laplace-Beltrami operator acting on function $\phi_e(\cdot,t)$ [3]. We remark that Eq. (S5), a damped wave equation, is exact under the assumption that $\Omega$ is unbounded [2], hence is an approximation since the physical cortical surface is bounded.

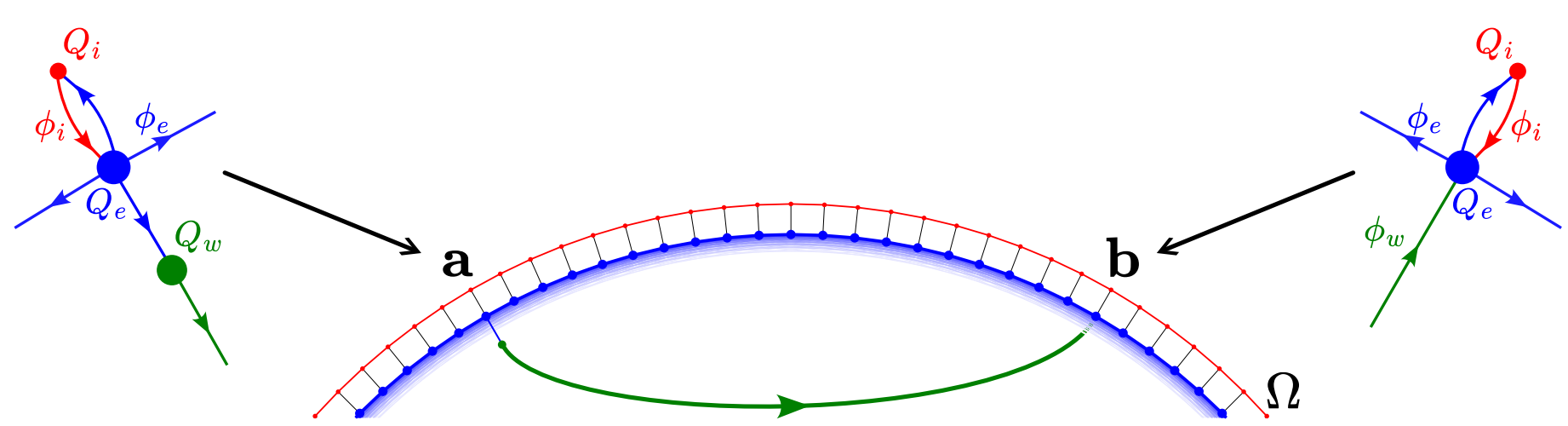


**Fig A. Schematic of physiological model.**

Complementing the propagator mechanisms of the model, are the synaptic mechanisms which map $\phi$ back to $Q$ at each position $\mathbf{r}$ and time $t$, illustrated in Fig. A. In summary, the synaptic activities of population type $e$ contribute to somatic activities of all population types ($\phi_e \to Q_e, Q_w, Q_i$), type $i$ contributes to types $e, i$ only ($\phi_i \to Q_e, Q_i$), and type $w$ contributes to type $e$ only ($\phi_w \to Q_e$). In addition, external input $f$ contributes to the somatic activities of type $e$. The choices of these interactions is to allow the total system to be reduced to a single equation, while capturing the following synaptic contributions: $\phi_e \to Q_w$ and $\phi_w \to Q_e$ for non-local connectomic propagation, $\phi_e \to Q_e$ for local geometric propagation, and $f \to Q_e$ for external input to drive the system. Mathematically, we used linear equations to represent the above synaptic mechanisms

$$Q_e(\mathbf{r},t) = G_{ee}(\mathbf{r})\phi_e(\mathbf{r},t) + G_{ei}(\mathbf{r})\phi_i(\mathbf{r},t) + G_{ew}(\mathbf{r})\phi_w(\mathbf{r},t) + f(\mathbf{r},t)\,; \tag{S8}$$

$$Q_w(\mathbf{r},t) = G_{we}(\mathbf{r})\phi_e(\mathbf{r},t)\,; \tag{S9}$$

$$Q_i(\mathbf{r},t) = G_{ie}(\mathbf{r})\phi_e(\mathbf{r},t) + G_{ii}(\mathbf{r})\phi_i(\mathbf{r},t)\,. \tag{S10}$$

In the equations above, each gain term $G_{ab}(\mathbf{r})$ is the change in $Q_a$ at position $\mathbf{r}$, per unit increase in $\phi_b$ at the same position and time. $G_{ab}$ is positive for $b = e, w$ and negative for $b = i$. Physiologically, this relationship arises from the pulses of the synapses of $b$ being 'transferred' to the dendrites of $a$ through neurotransmitter mechanisms, before arriving at the somas of $a$ [4]. The equations neglect the temporal spreading that arises from conducting delays within the dendritic trees of individual neurons, as these delays do not differ between locally and non-locally propagating populations $(e, w)$, thus out of the model's scope. The equations also assume the transfer of pulses is linear, which is a reasonable approximation if the model's variables are defined as first-order deviations about an assumed steady state [2]. The above Eqs (S5), (S6), (S7), (S8), (S9), (S10), when combined, reduces the system to a single non-local partial differential equation, with variable $\phi = \phi_e(\mathbf{r}, t)$, and zero initial conditions.

$$\left(1 - \tilde{G}_e(\mathbf{r}) + \frac{2}{\gamma}\frac{\partial}{\partial t} + \frac{1}{\gamma^2}\frac{\partial^2}{\partial t^2} - r^2\nabla^2\right)\phi(\mathbf{r}, t) = \tilde{G}_w(\mathbf{r})\phi(\mathbf{a}, t - \tau) + f(\mathbf{r}, t), \text{ where} \tag{S11}$$

$$\tilde{G}_e(\mathbf{r}) = G_{ee}(\mathbf{r}) + \frac{G_{ei}(\mathbf{r})G_{ie}(\mathbf{r})}{1 - G_{ii}(\mathbf{r})}, \text{ and } \tilde{G}_w(\mathbf{r}) = G_{ew}(\mathbf{b})G_{we}(\mathbf{b})\delta(\mathbf{r} - \mathbf{b})\,;$$
$$\phi(\mathbf{r}, 0) = \dot{\phi}(\mathbf{r}, 0) = 0\,. \tag{S12}$$

Finally we aim to calibrate the effective gain terms $\tilde{G}_e(\mathbf{r}), \tilde{G}_w(\mathbf{r})$ in space, to satisfy the property that, for any given input $f(\mathbf{r}, t)$, the total activity in the system integrated over space and time is preserved after the addition of population $w$. This preservation follows the approach through which neural field models are constructed, in which the total spacetime-integrated propagation of activity is preserved [5]. We let $\phi_0(\mathbf{r}, t)$ be the solution to the system in the absence of population $w$. Then, we have the constraint

$$\int_0^\infty dt \int_\Omega d^2\mathbf{r}\ \phi(\mathbf{r}, t) = \int_0^\infty dt \int_\Omega d^2\mathbf{r}\ \phi_0(\mathbf{r}, t). \tag{S13}$$

In the case of the geometric model, where population $w$ is absent, $\tilde{G}_e(\mathbf{r})$ is spatially uniform, and $\tilde{G}_w(\mathbf{r}) = 0$ for all $\mathbf{r}$. We let $\nu_0$ be the constant value of $\tilde{G}_e(\mathbf{r})$ in the absence of population $w$. Thus $\phi_0$ is the solution to the equation

$$\left(1 - \nu_0 + \frac{2}{\gamma}\frac{\partial}{\partial t} + \frac{1}{\gamma^2}\frac{\partial^2}{\partial t^2} - r^2\nabla^2\right)\phi_0(\mathbf{r}, t) = f(\mathbf{r}, t)\,; \tag{S14}$$

$$\phi_0(\mathbf{r}, 0) = \dot{\phi}_0(\mathbf{r}, 0) = 0\,. \tag{S15}$$

The local synaptic mechanisms of population $e$ in our model is identical at all points on $\Omega$ except $\mathbf{a}$, where synaptic activities of type $e$ contribute to population type $w$. Accordingly, we parameterize the effective gain term $\tilde{G}_e(\mathbf{r})$ to differ from $\nu_0$ at point $\mathbf{a}$ only, i.e.,

$$\tilde{G}_e(\mathbf{r}) = \nu_0 - c\,\delta(\mathbf{r} - \mathbf{a}), \tag{S16}$$

for some constant $c > 0$. Under the initial conditions in Eqs (S12), (S15), and the fact that $\int_\Omega d^2\mathbf{r}\ \nabla^2\phi(\mathbf{r}, t) = \int_\Omega d^2\mathbf{r}\ \nabla^2\phi_0(\mathbf{r}, t) = 0$ for all $t$ since $\Omega$ is a closed surface, integrating Eqs (S11), (S14) over space and time, and then applying constraint Eq. (S13), yields equality $G_{ew}(\mathbf{b})G_{we}(\mathbf{b}) = c$, implying

$$\tilde{G}_w(\mathbf{r}) = c\,\delta(\mathbf{r} - \mathbf{b}). \tag{S17}$$

Substituting Eqs (S16) and (S17) into Eq. (S11) yields the simplified equation:

$$\begin{aligned}&\left(1-\nu_0+\frac{2}{\gamma}\frac{\partial}{\partial t}+\frac{1}{\gamma^2}\frac{\partial^2}{\partial t^2}-r^2\nabla^2\right)\phi(\mathbf{r},t)\\&\qquad=-c\,\delta(\mathbf{r}-\mathbf{a})\,\phi(\mathbf{a},t)+c\,\delta(\mathbf{r}-\mathbf{b})\,\phi(\mathbf{a},t-\tau)+f(\mathbf{r},t)\,,\\&\phi(\mathbf{r},0)=\dot{\phi}(\mathbf{r},0)=0\,.\end{aligned}$$

Finally, we generalize the equation to multiple FNPs, where the $m$th FNP has source and term $\mathbf{a}_m, \mathbf{b}_m$, and connectivity parameter $c_m$. Accordingly, we attain the equation:

$$\mathcal{D}_t\phi(\mathbf{r},t)-r^2\nabla^2\phi(\mathbf{r},t)-(C\circ\phi)(\mathbf{r},t)=f(\mathbf{r},t)\,,\ \text{where} \tag{S18}$$

$$\mathcal{D}_t\phi(\mathbf{r},t)=\left(1-\nu_0+\frac{2}{\gamma}\frac{\partial}{\partial t}+\frac{1}{\gamma^2}\frac{\partial^2}{\partial t^2}\right)\phi(\mathbf{r},t)\,, \tag{S19}$$

$$\left(C\circ\phi\right)(\mathbf{r},t)=\sum_m c_m\big[\delta(\mathbf{r}-\mathbf{b}_m)\phi(\mathbf{a}_m,t-\tau_m)-\delta(\mathbf{r}-\mathbf{a}_m)\phi(\mathbf{a}_m,t)\big]\,; \tag{S20}$$

$$\phi(\mathbf{r},0)=\dot{\phi}(\mathbf{r},0)=0\,.$$

## Section B: Model numerics

In this appendix, we detail the numerical methods used to solve the differential equation of the model used in this work. The equation can be found in Eq. (S18), (S19), (S20). The methods used below also assume the particular model specifications used in this work: that the spatial domain is a square of length $L$ with periodic boundary conditions ($\Omega = (0, L]^2$), and the conduction time of the $m$th FNP is zero ($\tau_m = 0$) for all $m$.

For accessibility, we first rewrite the differential equation to separate the temporal derivative terms from other terms:

$$\phi(\mathbf{r}, t) + \frac{2}{\gamma}\dot{\phi}(\mathbf{r}, t) + \frac{1}{\gamma^2}\ddot{\phi}(\mathbf{r}, t) = P(\mathbf{r}, t)\,, \text{ for all } (\mathbf{r}, t) \in \Omega \times [0, \infty)\,, \text{ where} \tag{S21}$$
$$P(\mathbf{r}, t) = \nu_0\phi(\mathbf{r}, t) + r^2\nabla^2\phi(\mathbf{r}, t) + (C \circ \phi)(\mathbf{r}, t) + f(\mathbf{r}, t)\,;$$
$$\phi(\mathbf{r}, 0) = \dot{\phi}(\mathbf{r}, 0) = 0\,.$$

From $\Omega$, we uniformly partitioned a $N_x \times N_x$ computational grid of points with grid spacing $\Delta x = L/N$:

$$\Omega^* = \{\mathbf{r}_{ij} : i, j = 1, \ldots, N_x\} \subset \Omega, \text{ where } \mathbf{r}_{ij} = (i\Delta x, j\Delta x)\,. \tag{S22}$$

For the sake of computer simulation, the time domain is truncated to a bounded interval $[0, T]$, from which we uniformly partitioned $1 + N_t$ time points $\{t_n = n\Delta t : n = 0, 1, \ldots N_t\}$ with a time step $\Delta t = T/N_t$.

We define $\phi_{ij}^n = \phi(\mathbf{r}_{ij}, t_n)$ and $P_{ij}^n = P(\mathbf{r}_{ij}, t_n)$ as the numerical solutions at each grid point and time step. At each time step $n$, the numerical scheme we outline below computes $P_{ij}^n$ for $i, j$, and uses this value to compute $\phi_{ij}^{n+1}$ for all $i, j$.

$P_{ij}^n$ is computed on the computational grid as the sum of: $r^2\nabla^2(\mathbf{r}_{ij}, t_n)$, the local term; $(C \circ \phi)(\mathbf{r}_{ij}, t_n)$, the non-local term, and; $f(\mathbf{r}_{ij}, t_n)$, the external input. The first term, the local term, is approximated by the centered finite difference (five-point stencil), while incorporating the periodic boundary conditions of $\Omega$:

$$r^2\nabla^2\phi(\mathbf{r}_{ij}, t_n) \approx B_{ij}^n = \frac{r^2}{(\Delta x)^2}\left(\phi_{i+1,j}^n + \phi_{i,j+1}^n + \phi_{i-1,j}^n + \phi_{i,j-1}^n - 4\phi_{ij}^n\right)\,, \tag{S23}$$
$$\phi_{N+1,j}^n = \phi_{1,j}^n,\ \phi_{0,j}^n = \phi_{N,j}^n,\ \phi_{i,N+1}^n = \phi_{i,1}^n,\ \phi_{i,0}^n = \phi_{i,N}^n\,.$$

The second term, the non-local term, is computed as

$$\left(C \circ \phi\right)(\mathbf{r}_{ij}, t_n) = \sum_m c_m\big[\delta(\mathbf{r}_{ij} - \mathbf{b}_m) - \delta(\mathbf{r}_{ij} - \mathbf{a}_m)\big]\phi(\mathbf{a}_m, t_n).$$

since $\tau_m = 0$ for all $m$. Due to discontinuities of the delta terms $\delta(\mathbf{r}_{ij} - \mathbf{a}_m), \delta(\mathbf{r}_{ij} - \mathbf{b}_m)$ at $\mathbf{a}_m, \mathbf{b}_m$ respectively, and the possibility that the pointwise evaluation $\phi(\mathbf{a}_m, t_n)$ may not be attainable due to discretization, $(C \circ \phi)(\mathbf{r}_{ij}, t_n)$ is numerically computed with mollifiers, which are smooth functions that approximate the delta function. We first rewrite the term as

$$\left(C \circ \phi\right)(\mathbf{r}_{ij}, t_n) = \lim_{\epsilon \to 0}\sum_m c_m\left[k_\epsilon(\mathbf{r}_{ij} - \mathbf{b}_m) - k_\epsilon(\mathbf{r}_{ij} - \mathbf{a}_m)\right]\int_\Omega d^2\mathbf{r}' k_\epsilon(\mathbf{r}' - \mathbf{a}_m)\phi(\mathbf{r}', t_n)$$

where $k_\epsilon$ is a mollifier of parameter $\epsilon$ with unit integral over $\Omega$. For our experiments we approximate $(C \circ \phi)(\mathbf{r}_{ij}, t_n)$ by setting the mollifers $k_\epsilon$ as Gaussian in space, and

approximating the resultant integral by the Riemann sum expression:

$$(C \circ \phi)(\mathbf{r}_{ij}, t_n) \approx C^n_{ij} = \sum_{m=1} \frac{c_m}{(\Delta x)^2} \left[ w^{\mathbf{b}_m}_{ij} - w^{\mathbf{a}_m}_{ij} \right] \sum_{i',j'} w^{\mathbf{a}_m}_{i'j'} \phi^n_{i'j'}, \text{ where} \tag{S24}$$
$$w^{\mathbf{a}_m}_{ij} \propto \exp\left( \frac{-\|\mathbf{r}_{ij} - \mathbf{a}_m\|^2}{2\epsilon^2} \right), \; w^{\mathbf{b}_m}_{ij} \propto \exp\left( \frac{-\|\mathbf{r}_{ij} - \mathbf{b}_m\|^2}{2\epsilon^2} \right),$$
$$\sum_{i,j=1}^{N_x} w^{\mathbf{a}_m}_{ij} = \sum_{i,j=1}^{N_x} w^{\mathbf{b}_m}_{ij} = 1\,.$$

The third term, the external input term, is computed differently depending on whether the spatiotemporal profile of $f(\mathbf{r}, t)$ is an impulse-like input ($f^{\mathrm{stim}}(\mathbf{r}, t)$ in Eq. (2) of the main text) used to simulate stimulus-evoked responses, or a continuous space-time stochastic process ($f^{\mathrm{noise}}(\mathbf{r}, t)$ in Eq. (3) of the main text) used to simulate noise-driven activity. In the first case, the input term is pointwise evaluated at each grid point and timestep:

$$f(\mathbf{r}_{ij}, t_n) \approx f^{n,\mathrm{stim}}_{ij} \propto \exp\left( \frac{-\|\mathbf{r}_{ij} - \mathbf{r}_{os}\|^2}{2\sigma_x^2} \right) \exp\left( \frac{-(t_n - t_{os})^2}{2\sigma_t^2} \right), \tag{S25}$$
$$\sum_{n=0}^{1+N_t} \sum_{i,j=1}^{N_x} f^{n,\mathrm{stim}}_{ij} = \frac{1}{(\Delta t)(\Delta x)^2}\,.$$

In the second case, the white noise process $\dot{W}$ is treated as a piecewise constant random process [6], whereas the deterministic function $g(\mathbf{r})$ is pointwise evaluated at each grid point:

$$f(\mathbf{r}_{ij}, t_n) \approx f^{n,\mathrm{noise}}_{ij} = g_{ij} \frac{1}{\sqrt{\Delta t} \Delta x} \eta_{ij}\,, \tag{S26}$$
$$g_{ij} = g(\mathbf{r}_{ij})\,, \; \eta_{ij} \sim \mathcal{N}(0, 1)\,,$$

where $\mathcal{N}$ is the normal distribution.

The above Eqs (S23), (S24), (S25), (S26) are used to compute $P^n_{ij}$. This value is then used to compute $\phi^{n+1}_{ij}$ from $\phi^n_{ij}$ and $\phi^{n-1}_{ij}$. In this computation, the temporal derivatives in Eq. (S21) are approximated by centered finite differences:

$$\ddot{\phi}(\mathbf{r}_{ij}, t_n) \approx \frac{\phi^{n+1}_{ij} - 2\phi^n_{ij} + \phi^{n-1}_{ij}}{(\Delta t)^2}\,, \; \dot{\phi}(\mathbf{r}_{ij}, t_n) \approx \frac{\phi^{n+1}_{ij} - \phi^{n-1}_{ij}}{2(\Delta t)}\,.$$

In the case where $n = 0$, we substitute the initial conditions $\phi(\mathbf{r}, 0) = \dot{\phi}(\mathbf{r}, 0) = 0$ into the above finite difference approximations to obtain the approximations $\ddot{\phi}(\mathbf{r}_{ij}, t_0) = 2\phi^1_{ij}(\Delta t)^{-2}\,, \; \dot{\phi}(\mathbf{r}_{ij}, t_0) = 0\,.$

Put together, the numerical scheme used for the model is shown below:

$$\alpha = \frac{(\gamma \Delta t)^2}{2}; \; \beta_1 = \frac{(\gamma \Delta t)^2}{\gamma \Delta t + 1}; \; \beta_2 = \frac{2 - (\gamma \Delta t)^2}{\gamma \Delta t + 1}; \; \beta_3 = \frac{\gamma \Delta t - 1}{\gamma \Delta t + 1}; \tag{S27}$$
$$\forall i, j = 1, \dots N_x : \phi^0_{ij} = 0;$$
$$\forall n = 0, 1, \dots, N_t - 1 :$$
$$\quad \forall i, j = 1, \dots N_x : P^n_{ij} = \nu_0 \phi^n_{ij} + B^n_{ij} + C^n_{ij} + f^n_{ij}; \;\; \text{(S23), (S24), (S25), (S26)}$$
$$\quad \forall i, j = 1, \dots N_x : \phi^{n+1}_{ij} = \begin{cases} \alpha P^0_{ij}, \; n = 0, \\ \beta_1 P^n_{ij} + \beta_2 \phi^n_{ij} + \beta_3 \phi^{n-1}_{ij}, \; n \geq 1\,. \end{cases}$$

The values of the quantities introduced above for numerical treatment, $N_x, T, N_t, \epsilon$, used for our investigations are shown in Table A. $N_x$ is fixed to the value 200 to finely resolve radial traveling waves at millimeter order spatial resolution ($\Delta x = 0.4$ m$/200 = 2$ mm). The default value of $T$ was fixed to 70 ms to simulate the time-course of stimulus-evoked responses over short millisecond timescales. $N_t$ is fixed to the value 988 to satisfy the Courant–Friedrichs–Lewy condition of the two-dimensional wave equation ($\Delta x/\Delta t > r\gamma\sqrt{2}$) [7]. $\epsilon$ was fixed to 0.002 m, which was small enough for the mollifer $k_\epsilon$ to mimic the delta functional without encountering spurious oscillations in the spontaneous experiments in Fig. 5 of the main text.

**Table A. Quantities for numerical treatment**

| **Quantity** | **Value** |
|---|---|
| $N_x$ | 200 |
| $T$ | 0.07 s |
| $N_t$ | 988 |
| $\epsilon$ | 0.002 m |

## Section C: Algorithmic details

**Computation of BOLD responses.** Given the model's evoked response $\phi(\mathbf{r}, t)$ to an impulse stimulus under a given structural connectivity, the BOLD response (BOLD signal observation of the stimulus-evoked response) is approximated by $z(\mathbf{r})$, the zero temporal frequency component of $\phi(\mathbf{r}, t)$:

$$z(\mathbf{r}) = \mathcal{F}[\phi](\mathbf{r}, \omega = 0) = \int_0^\infty dt\ \phi(\mathbf{r}, t). \tag{S28}$$

Here, $\mathcal{F}$ is the Fourier transform as an operator on $\phi$ as a function of $t$ only. $z(\mathbf{r})$ is computed numerically at each grid point $\mathbf{r}_{ij}$ by computing the matrix $Z = (z(\mathbf{r}_{ij}))_{ij}$. $Z$ is computed as the matrix series

$$Z = \Delta t \sum_{n=1}^{\infty} \Phi[n]\,, \tag{S29}$$

where $\Phi[n] = \left(\phi_{ij}^n\right)_{ij}$. To compute the above series, we iteratively compute the partial sum

$$Y[m] = \Delta t \sum_{n=0}^{m \cdot N_t} \Phi[n] \text{ for } m = 1, 2, \ldots$$

We stop the iteration when the cosine distance between the vectors $\mathrm{vec}(Y[m]), \mathrm{vec}(Y[m-1])$, the vectorizations of the matrices $Y[m], Y[m-1]$, is less than the threshold $\mathrm{tol} = 10^{-5}$. The last computed $Y[m]$ is taken as an approximate scalar multiple of $Z$, since the cosine distance between $\mathrm{vec}(Y[m])$ and $\mathrm{vec}(Z)$ is close to zero. To compute $Z$ from $Y_m$, we use the fact that, by integrating both sides of Eq. (S18) over both space $\Omega$ and time $[0, \infty)$, one obtains

$$\begin{aligned} (1-\nu_0)\left(\int_\Omega d^2\mathbf{r}\ z(\mathbf{r})\right) &= \int_0^\infty dt \int_\Omega d^2\mathbf{r}\ \delta(\mathbf{r}-\mathbf{r}_0)\delta(t-t_0) = 1 \\ \implies \int_\Omega d^2\mathbf{r}\ z(\mathbf{r}) &= (1-\nu_0)^{-1}. \end{aligned}$$

Accordingly, $Z$ is computed by scalar multiplying $Y[m]$ so that the sum of all elements in $Z$ is $(\Delta x)^{-2}(1-\nu_0)^{-1}$.

**Computation of cosine dissimilarity metrics.** The cosine dissimilarity between the stimulus-evoked dynamics of the geometric model and any hybrid model containing additional FNPs, in response to the same impulse stimulus, is defined by

$$C_\phi(t) = 1 - \frac{\int_\Omega d^2\mathbf{r}\ \phi(\mathbf{r}, t)\phi_0(\mathbf{r}, t)}{\sqrt{\int_\Omega d^2\mathbf{r}\,(\phi(\mathbf{r}, t))^2}\sqrt{\int_\Omega d^2\mathbf{r}\,(\phi_0(\mathbf{r}, t))^2}}\,, \tag{S30}$$

where $\phi_0$ and $\phi$ are the geometric model's and hybrid model's activity respectively. Similarly, the cosine dissimilarity between the evoked BOLD response of the geometric model and any hybrid model containing additional FNPs, in response to the same impulse stimulus, is defined by

$$C_z = 1 - \frac{\int_\Omega d^2\mathbf{r}\ z(\mathbf{r}) z_0(\mathbf{r})}{\sqrt{\int_\Omega d^2\mathbf{r}\,(z(\mathbf{r}))^2}\sqrt{\int_\Omega d^2\mathbf{r}\,(z_0(\mathbf{r}))^2}}\,, \tag{S31}$$

where $z_0$ and $z$ are the geometric model's and hybrid model's BOLD responses respectively. $C_\phi(t)$ is used in our investigations to quantify the perturbation of an FNP, or a connectome of multiple FNPs on the model's stimulus-evoked dynamics over time, and $C_z$ quantifies the perturbation on the evoked BOLD response. To compute $C_\phi(t_n)$, where $t_n = n\Delta t$ is a timestep in the simulation, we computed the cosine distance between the vectors $\mathrm{vec}(\Phi[n])$ and $\mathrm{vec}(\Phi_0[n])$, where $\Phi[n] = \left(\phi(\mathbf{r}_{ij}, t_n)\right)_{ij}, \Phi_0[n] = \left(\phi_0(\mathbf{r}_{ij}, t_n)\right)_{ij}$. Similarly, $C_z$ is computed as the cosine distance between $\mathrm{vec}(Z)$ and $\mathrm{vec}(Z_0)$, where $Z = \left(z(\mathbf{r}_{ij})\right)_{ij}, Z_0 = \left(z_0(\mathbf{r}_{ij})\right)_{ij}$.

**Computation of Spatial Correlation Function.** The spatial correlation function with reference point $\mathbf{p}$, denoted $\gamma_{\mathbf{p}} : \Omega \to [-1, 1] : \mathbf{r} \to \mathrm{corr}(\phi(\mathbf{p}, t), \phi(\mathbf{r}, t))$, returns the Pearson correlation between the model's noise-driven activity at point $\mathbf{p}$ and any input point $\mathbf{r}$ on $\Omega$. In our investigations, we numerically compute $\gamma_{\mathbf{p}}$ evaluated at all grid points $\mathbf{r}_{ij}$ by computing the matrix $\Gamma = \left(\gamma_{\mathbf{p}}(\mathbf{r}_{ij})\right)_{ij}$. Each element of $\Gamma$ is computed as

$$\gamma_{\mathbf{p}}(\mathbf{r}_{ij}) = \lim_{m\to\infty} r_{ij}[m],$$

$$r_{ij}[m] = \frac{\frac{\sum_{n=1}^{m\cdot N_t} \phi_{\mathbf{p}}^n \phi_{ij}^n}{m\cdot N_t} - \frac{\sum_{n=1}^{m\cdot N_t} \phi_{\mathbf{p}}^n}{m\cdot N_t} \cdot \frac{\sum_{n=1}^{m\cdot N_t} \phi_{ij}^n}{m\cdot N_t}}{\sqrt{\frac{\sum_{n=1}^{m\cdot N_t} \left(\phi_{\mathbf{p}}^n\right)^2}{m\cdot N_t} - \left(\frac{\sum_{n=1}^{m\cdot N_t} \phi_{\mathbf{p}}^n}{m\cdot N_t}\right)^2}\sqrt{\frac{\sum_{n=1}^{m\cdot N_t} \left(\phi_{ij}^n\right)^2}{m\cdot N_t} - \left(\frac{\sum_{n=1}^{m\cdot N_t} \phi_{ij}^n}{m\cdot N_t}\right)^2}},$$

where $\phi_{\mathbf{p}}^n$ is the simulated time series $\phi(\mathbf{r}, t_n)$ at point $\mathbf{p} = (0.15, 0.15)$. For grid size $N_x = 200$ and domain length $L = 0.4$, $\phi_{\mathbf{p}}^n$ is the time series $\phi_{75,75}^n$. Iteratively with $m = 1, 2, \ldots$, we compute the matrix $R[m] = (r_{ij}[m])_{ij}$. We stop the iteration when the correlation between the vectors $\mathrm{vec}(R[m]), \mathrm{vec}(R[m-1])$ is less than the threshold $\mathrm{tol} = 10^{-7}$. The last attained $R[m]$ is then taken as the numerical computation of $\Gamma$.

**Sampling of connectomes with non-random properties** Connectomes constrained by non-random properties (exponential distance rule, hub specificity and rich-club specificity) are sampled using a rejection-sampling algorithm. The source and target point of each FNP is sampled uniformly and independently on $\Omega$, but is accepted only if the sampled source and target passes a acceptance-rejection test tailored to the non-random property under investigation; otherwise the source and target is sampled again. Once the sampled source and target is accepted, the next FNP is sampled in a similar manner. We emphasize, however, that each connectome we generate is constrained by one of the three non-random properties only.

**Acceptance–rejection test for exponential distance rule (EDR) constraint.** To sample an FNP with parameter $\lambda_e$, a source $\mathbf{r}_1$ and target $\mathbf{r}_2$ is first sampled uniformly from $\Omega$. A value $u$ is then sampled from the Uniform$[0, 1]$ distribution. $\mathbf{r}_1$ and $\mathbf{r}_2$ are accepted if $u < \exp(-100\lambda_e l)$, where $\lambda_e \in [0, 1]$ is the selected decay parameter, and $l$ is the Euclidean distance between the source and target (with periodic boundary conditions). Otherwise, $\mathbf{r}_1$, $\mathbf{r}_2$ and $u$ are resampled. In this way, the probability that a FNP exists with source $\mathbf{r}_1$ and target $\mathbf{r}_2$, for any two given points $\mathbf{r}_1$ and $\mathbf{r}_2$, is

$$\mathbb{P}\left(\text{FNP } \mathbf{r}_1 \to \mathbf{r}_2 \mid \mathbf{r}_1, \mathbf{r}_2\right) = \exp\left(-100\lambda_e \|\mathbf{r}_1 - \mathbf{r}_2\|\right). \tag{S32}$$

**Acceptance–rejection test for hub specificity constraint.** To sample an FNP with parameter $\lambda_h$, a value $u$ is first sampled from the Uniform$[0, 1]$ distribution. If $u < \lambda_h$, the source and target points of the FNP are repetitively sampled uniformly from $\Omega$ and accepted when the FNP is hub-connecting, i.e., the FNP connects a point

inside any one hub with a point outside that hub. Otherwise, if $u \geq \lambda_h$, the source and target points are sampled once and accepted. In this way, the probability that any given FNP is hub-connecting is

$$\mathbb{P}\,(\text{FNP is hub-connecting}) = (1-\lambda_h)p_h + \lambda_h\,, \tag{S33}$$

where $p_h$ is the probability that a uniformly sampled FNP is hub-connecting. As $\lambda_h$ increases from 0 to 1, $\mathbb{P}\,(\text{FNP is hub-connecting})$ increases from $p_h$ to 1. Theoretically,

$$p_h = 1 - n\left(\frac{|\Omega_h|}{|\Omega|}\right)^2 - \left(1 - n\frac{|\Omega_h|}{|\Omega|}\right)^2\,,$$

where $|\Omega_h|$ is the area of each hub, $n$ is the number of hubs, and $|\Omega|$ is the area of $\Omega$. For our investigations ($n = 4, |\Omega_h| = |\Omega|/34$), $p_h \approx 0.22$. $u$ is then resampled for the next FNP.

**Acceptance–rejection test for rich-club specificity constraint.** To sample an FNP with parameter $\lambda_r$, a value $u$ is first sampled from the Uniform$[0,1]$ distribution. If $u < \lambda_r$, the source and target points of the FNP are repetitively sampled uniformly from $\Omega$ and accepted when the FNP is a rich-club connection, i.e., it connects a point inside any one hub with a point inside another hub. Otherwise, if $u \geq \lambda_r$, the source and target points are sampled once and accepted. In this way, the probability that any given FNP is hub-connecting is

$$\mathbb{P}\,(\text{FNP is a rich-club connection}) = (1-\lambda_r)p_r + \lambda_r\,, \tag{S34}$$

where $p_r$ is the probability that a uniformly sampled FNP is a rich-club connection. As $\lambda_r$ increases from 0 to 1, $\mathbb{P}\,(\text{FNP is a rich-club connection})$ increases from $p_r$ to 1. Theoretically,

$$p_r = n(n-1)\left(\frac{|\Omega_h|}{|\Omega|}\right)^2\,,$$

where $|\Omega_h|$ is the area of each hub, $n$ is the number of hubs, and $|\Omega|$ is the area of $\Omega$. For our investigations ($n = 4, |\Omega_h| = |\Omega|/34$), $p_r \approx 0.01$. $u$ is then resampled for the next FNP.

# References


1. Nunez PL, Cutillo BA, editors. Neocortical Dynamics and Human EEG Rhythms. New York: Oxford University Press; 1995.

2. Robinson PA, Rennie CJ, Wright JJ. Propagation and Stability of Waves of Electrical Activity in the Cerebral Cortex. Physical Review E. 1997;56(1):826–840. doi:10.1103/PhysRevE.56.826.

3. Sario L, Nakai M, Wang C, Chung LO. Laplace-Beltrami Operator. In: Sario L, Nakai M, Wang C, Chung LO, editors. Classification Theory of Riemannian Manifolds: Harmonic, Quasiharmonic and Biharmonic Functions. Berlin, Heidelberg: Springer; 1977. p. 12–25.

4. Robinson PA. Propagator Theory of Brain Dynamics. Physical Review E. 2005;72(1):011904. doi:10.1103/PhysRevE.72.011904.

5. Robinson PA. Near-Critical Corticothalamic Eigenmodes: Effects of Nonuniform Connectivity on Modes, Activity, and Communication Channels. Physical Review E. 2025;111(1):014404. doi:10.1103/PhysRevE.111.014404.

6. Allen EJ, N S J, and Zhang Z. Finite Element and Difference Approximation of Some Linear Stochastic Partial Differential Equations. Stochastics and Stochastic Reports. 1998;64(1-2):117–142. doi:10.1080/17442509808834159.

7. Lines LR, Slawinski R, Bording RP. A Recipe for Stability of Finite-difference Wave-equation Computations. Geophysics. 1999;64(3):967–969. doi:10.1190/1.1444605.
