# Supplementary material for: Modeling the influences of non-local connectomic projections on geometrically constrained cortical dynamics
Source: PLoS Comput Biol. 2026 Aug 26;22(8):e1014673. doi: 10.1371/journal.pcbi.1014673 (PMC13552967; doi:10.1371/journal.pcbi.1014673)
Supplement: Supplementary file 6 [file pcbi.1014673.s006.pdf]

Review of "Modeling the influences of non-local connectomic projections on geometrically constrained cortical dynamics"

The article "Modeling the influences of non-local connectomic projections on geometrically constrained cortical dynamics" by Maran et al analyzes, from a mathematical modeling standpoint, the competing effects of long-range versus local-geometric cortical activity propagation.

The manuscript is scientifically solid and generally well-structured. It provides valuable and interesting insights into the propagation of cortical activity within a mixed continuum/networked structure, which is modeled through a simplified framework. In particular, I appreciate how the authors try to interpret the issue of experimental detectability of Fast-conducting Non-local Projections (FNPs). While the model employed resembles earlier approaches, the analysis presented in the manuscript is both novel and comprehensive. Moreover, the authors have already addressed the concerns previously raised by other Reviewers for PLOS Biology.

Overall, I am convinced of the relevance of this work to theoretical and computational neuroscience, and I consider it suitable for publication in PLOS Computational Biology. However, I have a few minor remarks regarding the code documentation and the presentation of the model, and a specific comment about part of the results that the authors should address before publication.

Code documentation

I note positively the free availability of the code necessary to reproduce numerical experiments. My only comment here is that the readme file within the code repository is currently too succinct. To be useful, it should report (i) a brief description of the model, (ii) the code structure, (iii) software/language/package version information. For instance, although I am aware that GitHub automatically detects the language(s) used in a repository, I found it odd that neither the paper nor the readme ever mentioned that the code is written in Matlab, which suggests that the code description should be revised and expanded. I would also recommend cross-referencing the article, provisionally in its preprint form, using the corresponding DOI.

Model presentation

While using the shorthand $\mathcal{D}_t$ in eq. (1) is appropriate, the explicit form of the differential operator should appear in the main text. Including it in the section 'Model Implementation' seems an adequate solution to this omission.

On a similar note, the choice of the symbol r to indicate the characteristic length scale of geometric connectivity is inconvenient, as it can cause some visual confusion for the reader due to similarity with the spatial coordinates, $\boldsymbol{r}$. I suggest changing the name of this constant, e.g. to $\boldsymbol{\ell}$.

Dynamical correlation under white noise stimuli

In Fig. 5, you analyze spatial correlations of neural activity in the presence of a single FNP. Later (Fig. 6) you examine the effect of including many FNPs in the response to a pulse stimulus by means of the dissimilarity C(t). To bridge these results, I would suggest to try and extract some information on spatio-temporal correlations of activity under white noise stimuli, e.g. as described by $G(|r-r'|, |t-t'|) = \langle \phi(r,t)\phi(r',t') \rangle$, where averaging here is intended both over noise and over space and time, at stationarity. In this sense, two possible suggestions are:

- Plot the temporal and spatial correlation scales (extracted from G) as a function of N (the number of FNPs) finely varying N over a few orders of magnitudes (range $\{1, \dots, 1000\}$ should be enough)
- Plot a heatmap of $G(\Delta r, \Delta t)$ for a few choices of N (e.g. N=1,10,100,1000)

Unless I am missing something, this kind of analysis should produce relevant information not currently present in the paper. Also, I don't believe it is numerically too expensive, with the only clear bottleneck being the resampling of the FNPs configuration (here, completely random FNPs should suffice). Together, such two figures could strengthen the core of this analysis by characterizing the baseline response properties of the system. Even in the case that nothing unexpected emerges, they would make for a solid addition to the Supporting Information.

Other minor comments

- Found an unmatched parenthesis on line 138
- In Fig. 5 B, fixing the colorbar boundary values would make for easier comparisons across subpanels.
