# Supplementary material for: Modeling the influences of non-local connectomic projections on geometrically constrained cortical dynamics
Source: PLoS Comput Biol. 2026 Aug 26;22(8):e1014673. doi: 10.1371/journal.pcbi.1014673 (PMC13552967; doi:10.1371/journal.pcbi.1014673)
Supplement: Supplementary file 7 [file pcbi.1014673.s008.pdf]

We thank the editor and reviewers for their thoughtful comments, feedback, and suggestions. This document contains a point-by-point response to all reviewer comments in full (shown in *dark blue italics*). Edits to the manuscript text are shown in boxes, and where edits are mixed with existing quoted text from our original manuscript, the edits have been distinguished using boldface. Quoted text from the manuscript is shown as LaTeX source.

Reviewer #1

Point 1.1

The manuscript presents a clear and interesting theoretical contribution to the ongoing debate on the respective roles of cortical geometry and long-range connectivity in shaping macroscale brain dynamics. The authors introduce a hybrid neural field framework that combines geometrically constrained propagation on a continuous cortical sheet with discrete fast non-local projections, and they use this model to study how the contribution of these two mechanisms depends on timescale and input conditions. Overall, I enjoyed reading the manuscript, wich is well written, mathematically clear, and conceptually relevant. In particular, the main result — that fast non-local projections strongly affect rapid stimulus-evoked dynamics but have a much weaker influence on slower spontaneous fluctuations — is compelling and provides a useful reconciliation between geometry-based accounts and connectome-based accounts of resting-state activities. I only have a few minor suggestions, as follows.

We thank the reviewer for their positive assessment of our manuscript.

Point 1.2

First, I think the manuscript would benefit from a slightly more restrained statement of novelty. The important contribution, in my view, is not the coexistence of local geometric propagation and non-local projections within one framework, but the explicit demonstration that their relative importance depends strongly on temporal regime and input structure (even if some of the assumptions might be irrealistic at this stage). Clearly framing the advancement in those terms would make the paper more precise and, in fact, stronger in my view. This is important because the paper proposes a framework that addresses a tension currently present in the literature.

We thank the reviewer for the suggestion, and agree that the key novelty of our manuscript is its contribution to resolving the relative role of connectomic and geometric mechanisms across timescales and input structures. While the model construction introduced here is indeed (to our knowledge) a new mathematical formulation of these mechanisms that we introduce for the first time, we have mostly removed the use of the adjectives "novel" and "new" when describing the model in order to better emphasize the key contributions of the manuscript to the scientific problem it focuses on addressing. For example, the key framing in the abstract now just mentions "a mathematical model":

> […]
> Here we address this question by conducting a range of investigations using **a mathematical model** of macroscale cortical activity, in which cortical populations interact both by a continuous sheet and by an additional set of FNPs wired independently of the sheet's geometry. […]

Point 1.3

Also, one potential improvement could come if the authors would make the connection to empirical neuroscience a little more. Firstly, I would suggest to consider this manuscript (The structural connectome constrains fast brain dynamics), based on empirical data, as it is directly relevant to the manuscript's central clain. Also, the paper could more explicitly highlight which experimental settings are most relevant to the model's predictions. In its current form, the manuscript already motivates the problem through fast evoked activity, resting-state fMRI, and related macroscale recordings, so this step would be quite natural.

We agree with the suggestion to emphasize connections to empirical measurement. The empirical findings of Sorrentino et al. e*Life* (2021), "*The structural connectome constrains fast brain dynamics"* are indeed central to one of the central claims of the manuscript; we have now incorporated this paper into our manuscript:

> Here we seek to mechanistically reconcile two seemingly conflicting empirical findings: (i) direct evidence of the role of FNPs in facilitating the non-local dynamics of stimulus-evoked whole-brain responses measured with voltage-sensitive dye (VSD) imaging \cite{ferezouSpatiotemporalDynamicsCortical2007, mohajeraniSpontaneousCorticalActivity2013, limVivoLargeScaleCortical2012}, electroencephalography (EEG) \cite{massiminiCorticalMechanismsLoss2012, seguinCommunicationDynamicsHuman2023}, **magnetoencephalography (MEG) \cite{sorrentino2021structural}**, and wide-field calcium imaging

\cite{liuCellClassspecificLongrange2024}; and (ii) the predictive successes of geometrically constrained neural field models, which ignore the specificity of FNP connectivity informed by connectomic data, in capturing the spatiotemporal dynamics of resting-state functional magnetic resonance imaging (fMRI) \cite{robinsonDeterminationDynamicBrain2021, hendersonEmpiricalEstimationEigenmodes2022, pangGeometricConstraintsHuman2023}, the spatial patterns of fMRI task activation maps \cite{hendersonEmpiricalEstimationEigenmodes2022, pangGeometricConstraintsHuman2023}, and resting-state magnetoencephalography (MEG) \cite{hendersonEmpiricalEstimationEigenmodes2022}.

Note that Sorrentino et al. (2021) analyzed resting-state (rather than stimulus-evoked) neural activity measured using MEG and, as such, it focused on the spatiotemporal properties of spreading processes evoked by focal suprathreshold perturbations, that occurred momentarily in the spontaneous regime. Given how spontaneous versus stimulus-evoked dynamics are treated in the mathematical model introduced in our manuscript, such spontaneously occurring spreading processes analyzed by Sorrentino et al. (2021) can be interpreted mathematically as stimulus-evoked responses. Hence, we think it's appropriate to cite this paper as evidence in support of the relevance of FNPs in stimulus-evoked MEG dynamics.

We also appreciate the suggestion to highlight the experimental settings that are most relevant to the model predictions. This is discussed in a dedicated *Discussion* paragraph, an excerpt from which is reproduced below:

[...]
The findings of this work suggest that the continuous geometric approximation of structural connectivity (akin to neural field models) becomes a stronger approximation for capturing the spatiotemporal patterns of spontaneous cortical dynamics on longer timescales (like that of resting-state fMRI), while the additional non-local specificity of the connectome (which can be incorporated in neural mass models) becomes more crucial for capturing the cortex's more rapid processing of spatially localized stimuli. Note that this does not imply that the specificity of the connectome is unimportant for capturing resting-state fMRI dynamics---indeed, the further constraining of geometric connectivity with specific connections from connectomic data can improve predictions of resting-state fMRI dynamics \cite{decoRareLongrangeCortical2021, vohryzekHumanBrainDynamics2025, decoNonlocalSchrodingerDiffusion2025}. Rather, our findings highlight stimulus-evoked response dynamics as an experimentally challenging but important regime for developing and refining future dynamical models of macroscale brain

activity — a regime in which the specificity of structural connectivity may play a more significant role than in resting-state fMRI \cite{maranAnalyzingBrainsDynamic2025}.

Point 1.4

In summary, this is a strong and thoughtful computational paper that addresses an important question with appropriate theoretical tools. I compliment the authors. The results are interesting, the manuscript is generally clear, and the main conclusions are worthy of publication in my opinion.

We once again thank the reviewer for their positive assessment and thoughtful suggestions that have allowed us to improve our manuscript.

Reviewer #2

Point 2.1

I reviewed the manuscript originally submitted to PLoS Biology. All reviewers concerned that the claims of biology relevance were not sufficiently supported as the modeling was based on simplifying assumptions. In this revised version submitted now to PLoS Computational Biology, the authors stressed that the model aimed to present two key phenomenological mechanisms: (i) geometric spreading as travelling waves; and (ii) rapid non-local connectomic propagation.. The authors expected similar qualitative behavior from a large class of specifically formulated models that contain the two high-level mechanisms that the paper (and phenomenological model) targets. While some properties of simulated dynamics of more complex models would better capture some complexities found in real data, the modeling results would be less clearly generalizable. Limitations of the models are more adequately discussed.

We thank the reviewer for their acknowledgement of the improvements made to the manuscript, regarding its framing and interpretation in particular, in response to the prior round of review.

Point 2.2

Following the same logic, however, I would like to point that the emphasized novel finding that "FNPs shape the model dynamics of rapid, stimulus-evoked responses on fine millisecond timescales ($\lessapprox$ 30 ms), but contribute relatively little to slower, spontaneous fluctuations over longer timescales (>30 ms)", may not be really generalizable, especially the quantitative (single) threshold of 30 ms, as this could be the result of the "specific" over-simplifying assumptions of the model (passive diffusion rather than the excitability property of the neural field, and simplified topology of FNP when compared to empirical connectomes). I would suggest that the authors make this point clearer already in Abstract, Introduction, not just in Discussion.

We agree that the specific value of 30 ms is approximate given our model formulation. Further, as mentioned in the prior round of review, we expect this value likely to vary substantially with the global brain state. To avoid misunderstanding, we have now removed this specific quantification ("~ 30 ms") and replaced it with more general descriptions of its order of magnitude ('of tens of millisconds') in the abstract and Author Summary.

Abstract:

[...]

By simulating the model across a range of external inputs, timescales, and idealized connectome topologies, we demonstrate that the addition of FNPs strongly shape the model dynamics of rapid, stimulus-evoked responses on fine timescales **of tens of milliseconds**, but contribute relatively little to slower, spontaneous fluctuations over longer **order-of-seconds** timescales, which increasingly resemble geometrically constrained dynamics without FNPs.
[...]

Author summary:

[...]
Simulations of this model across a range of conditions show that specific long-range projections are crucial for reproducing rapid, stimulus-evoked responses on short timescales **of tens of milliseconds**, but contribute relatively little to slower, spontaneous fluctuations **over longer timescales of the order of seconds**.
[...]

Point 2.3

Apart from this point, in my view, the revised version is satisfactory for PLoS CB.

We appreciate the reviewer's time and feedback that has allowed us to more accurately frame and interpret our modeling work.

Reviewer #3

Point 3.1

The article "Modeling the influences of non-local connectomic projections on geometrically constrained cortical dynamics" by Maran et al analyzes, from a mathematical modeling standpoint, the competing effects of long-range versus local-geometric cortical activity propagation.

The manuscript is scientifically solid and generally well-structured. It provides valuable and interesting insights into the propagation of cortical activity within a mixed continuum/networked structure, which is modeled through a simplified framework. In particular, I appreciate how the authors try to interpret the issue of experimental detectability of Fast-conducting Non-local Projections (FNPs). While the model employed resembles earlier approaches, the analysis presented in the manuscript is both novel and comprehensive. Moreover, the authors have already addressed the concerns previously raised by other Reviewers for PLOS Biology.

Overall, I am convinced of the relevance of this work to theoretical and computational neuroscience, and I consider it suitable for publication in PLOS Computational Biology. However, I have a few minor remarks regarding the code documentation and the presentation of the model, and a specific comment about part of the results that the authors should address before publication.

We appreciate the reviewer's positive feedback.

Point 3.2

Code documentation

I note positively the free availability of the code necessary to reproduce numerical experiments. My only comment here is that the readme file within the code repository is currently too succinct. To be useful, it should report (i) a brief description of the model, (ii) the code structure, (iii) software/language/package version information. For instance, although I am aware that GitHub automatically detects the language(s) used in a repository, I found it odd that neither the paper nor the readme ever mentioned that the code is written in Matlab, which suggests that the code description should be revised and expanded. I would also recommend cross-referencing the article, provisionally in its preprint form, using the corresponding DOI.

We thank the reviewer for this constructive feedback. We have added more detail to the repository's README file to ensure reproducibility and clarity. Specifically, we have:

- Added a dedicated model description section outlining the simulation of the 2D cortical sheet and FNPs.
- Documented the repository structure and explicitly stated that the code is written in MATLAB, noting the requirement for the Parallel Computing Toolbox.
- Provided clear, step-by-step instructions for adding the Model directory to the MATLAB path and running the experiment scripts.
- Added a cross-reference and direct DOI link to the *arXiv* preprint version of the article at the top of the file.

For reference, the repository is available at:

https://github.com/DynamicsAndNeuralSystems/geometricFNPmodel

Point 3.3

Model presentation

While using the shorthand D_t *in eq. (1) is appropriate, the explicit form of the differential operator should appear in the main text. Including it in the section 'Model Implementation' seems an adequate solution to this omission.*

We have now added the explicit form of the differential operator in the Model Description:

[...]
In Eq.~\eqref{eq:model}, **$\mathcal{D}_t = 1-\nu_0 + 2 \gamma^{-1} \partial_t + \gamma^{-2} \partial_{tt}$** is a second-order linear differential operator in time, and the term $r^2 \nabla^2 \phi(\mathbf{r}, t)$ captures local spatial relations on Ω for different points $\mathbf{r}$.
[...]

Point 3.4

On a similar note, the choice of the symbol r to indicate the characteristic length scale of geometric connectivity is inconvenient, as it can cause some visual confusion for the reader due to similarity with the spatial coordinates, r. I suggest changing the name of this constant, e.g. to ℓ.

While we agree there is a potential for confusion between *r* (denoting the characteristic lengthscale of geometric connectivity) and boldface ***r*** (for spatial coordinates), this is a standard notational choice in Neural Field Theory that has been used across the literature, from a foundational Robinson et al. 1997 paper[1] through to current work (e.g., Robinson 2025[2]). We assess that breaking with this established notational convention is likely to introduce more confusion, so have chosen to retain the *r* notation for the lengthscale and ***r*** for the spatial coordinates here.

[1] P.A. Robinson, C.J. Rennie, & J.J. Wright (1997). Propagation and stability of waves of electrical activity in the cerebral cortex. *Physical Review E*, **56**(1): 826.

[2] P. A. Robinson (2025). Near-critical corticothalamic eigenmodes: Effects of nonuniform connectivity on modes, activity, and communication channels. *Physical Review E*, **111**(1): 014404.

Point 3.5

Dynamical correlation under white noise stimuli

In Fig. 5, you analyze spatial correlations of neural activity in the presence of a single FNP. Later (Fig. 6) you examine the effect of including many FNPs in the response to a pulse stimulus by means of the dissimilarity C(t). To bridge these results, I would suggest to try and extract some information on spatio-temporal correlations of activity under white noise stimuli, e.g. as described by $G(|r - r'|, |t - t'|) = \langle \varphi(r, t)\varphi(r', t') \rangle$*, where averaging here is intended both over noise and over space and time, at stationarity. In this sense, two possible suggestions are:*

- *Plot the temporal and spatial correlation scales (extracted from G) as a function of N (the number of FNPs) finely varying N over a few orders of magnitudes (range {1, . . . , 1000} should be enough)*
- *Plot a heatmap of* $G(\Delta r, \Delta t)$ *for a few choices of N (e.g. N=1,10,100,1000)*

Unless I am missing something, this kind of analysis should produce relevant information not currently present in the paper. Also, I don't believe it is numerically too expensive, with the only clear bottleneck being the resampling of the FNPs configuration (here, completely random FNPs should suffice). Together, such two figures could strengthen the core of this analysis by characterizing the baseline response properties of the system. Even in the case that nothing unexpected emerges, they would make for a solid addition to the Supporting Information.

We thank the reviewer for this interesting suggestion. Unfortunately, for the hybrid connectivity case with FNPs, the function $G(\Delta r, \Delta t)$ may not be suitable to calculate, because any specific distribution of FNPs will be non-uniform in general, making $G(r,r', \Delta t = 0)$ anisotropic. Furthermore we found that if we computed $\bar{G}(\Delta r, 0)$ averaged across space, the averaging `washes out' the outlier correlations at the specific pairs of points mediated by the FNPs, and so $\bar{G}(\Delta r, \Delta t=0)$ turns out to be very similar between the geometric and hybrid (with FNPs) connectivities.

It was due to both of these reasons that we computed the spatial correlation function in Fig. 5 separately at each point $\boldsymbol{r}$, without averaging over space. Finally, when $\Delta t > 0$, making $\bar{G}$ a cross-correlation function, we found that as Δt increased, $\bar{G}(\Delta r, \Delta t)$ became more spatially homogeneous, i.e., $\bar{G}(\Delta r, \Delta t)$ approached a constant function of Δr as Δr increased. This was true for both the geometric connectivity and hybrid connectivity cases. As a result, comparing $\bar{G}(\Delta r, \Delta t)$ for geometric and hybrid connectivity for large Δt ($\Delta t >= 20$ milliseconds) did not provide any interesting insights as they were both similar constants functions.

To illustrate the findings above, we computed $\bar{G}$ for geometric connectivity, and average of all $\bar{G}$'s computed with 10 independently sampled hybrid connectivities, each with 100 uniformly sampled FNPs, as was done in Fig. 6. As shown below, $\bar{G}(\Delta r, 0)$ is largely spatially isotropic under hybrid connectivity, thus similar to $\bar{G}(\Delta r, 0)$ under geometric connectivity, which is consistent with our finding of geometry dominating dynamics under spontaneous settings. Also when Δt = 0.02 s, $\bar{G}(\Delta r, \Delta t)$ is largely spatially uniform under both geometric and hybrid connectivity. Code to generate this figure is available as "correlation_scale_spontaneous.m" in the *Experiments* folder in the *GitHub* repository.

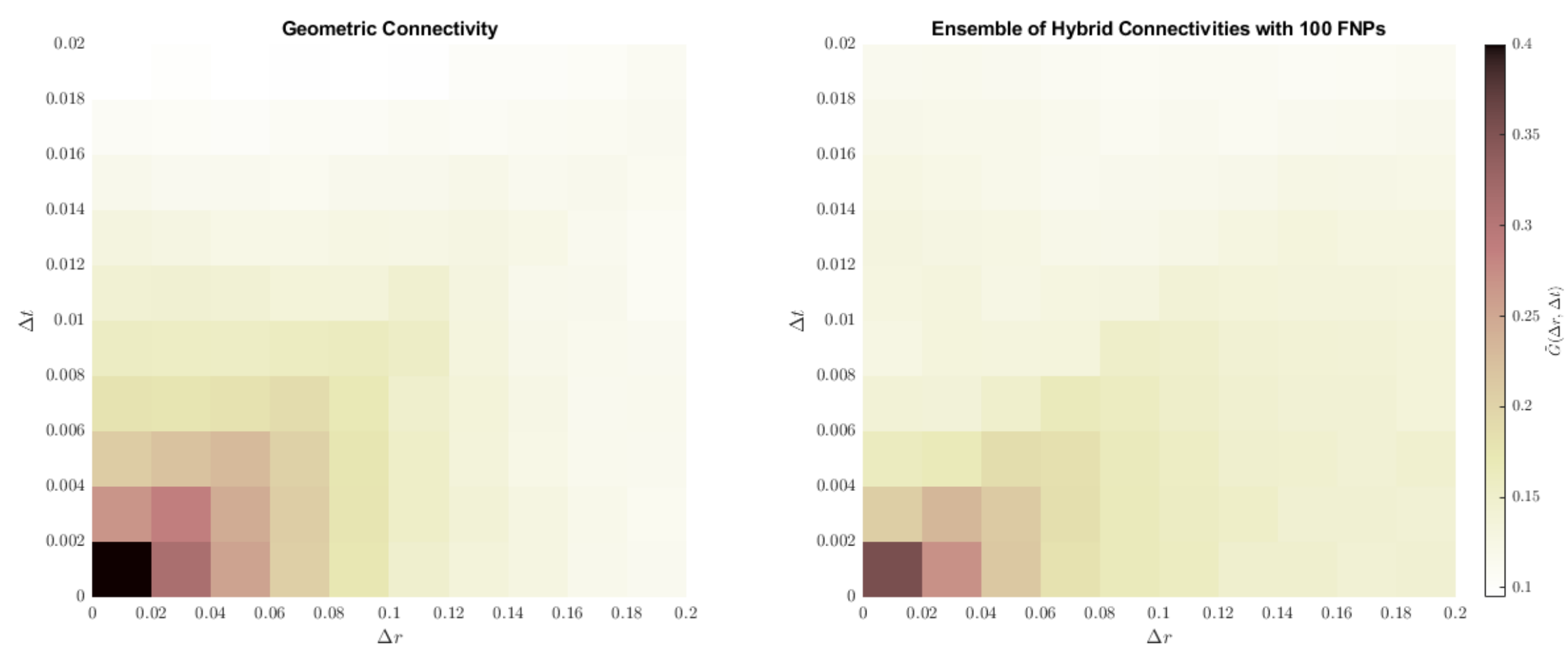

Point 3.6

Other minor comments

- *Found an unmatched parenthesis on line 138*
- *In Fig. 5 B, fixing the colorbar boundary values would make for easier comparisons across subpanels.*

We thank the reviewer for flagging these two issues; both have now been fixed in the revised manuscript.
