# Supplementary material for: Modeling the influences of non-local connectomic projections on geometrically constrained cortical dynamics
Source: PLoS Comput Biol. 2026 Aug 26;22(8):e1014673. doi: 10.1371/journal.pcbi.1014673 (PMC13552967; doi:10.1371/journal.pcbi.1014673)
Supplement: Supplementary file 9 [file pcbi.1014673.s007.docx]

This document contains a point-by-point response to all reviewer comments from this manuscript’s previous round of review at *PLoS Biology (PBIOLOGY-D-25-02558R1).* Where edits are mixed with existing quoted text from our original manuscript, the edits have been distinguished using boldface. Quoted text is shown as LaTeX source.

# **Reviewer #1**

***Point 1.1***

*The constituent axonal fibers (named fast-conducting non-local projections, FNPs) are indispensable in some experiments (e.g. millisecond-resolved voltage-sensitive recordings of stimulus responses) and are often ignored when treating the cortex as neural-field models on a continuous two-dimensional space on which activity spreads as travelling waves. To address the question that FNPs function diversely in different time scales, the authors build a mathematical model which contains both factors: local, geometry-constrained wave propagation along a cortical space and FNPs between disperse spatial points.*

*The cortex is represented by a continuous surface Ω (numerically chosen as a flat torus with a closed surface that matches human hemisphere). Neural activity at a millimeter-scale position r and time t is ϕ(r,t). The evolution of ϕ(r,t) obeys a non-local hyperbolic partial differential equation, which involves a second-order temporal operator D_t that supports finite-speed waves, the scale parameter r of local connectivity, the external input f(r,t) and (C∘ϕ)(r,t). The specific term (C∘ϕ)(r,t) is the sum over FNPs: each projection instantaneously propagates activity from its source point to the target point with connectivity strength c_m. Conduction delays τ_m are set to zero to emphasise the rapidity of FNP transmission relative to wave propagation.*

*The authors conduct several numerical experiments, ranging from single FNP to network of FNPs with random or inhomogeneous topology (hub and rich club). From these numerical simulations, the authors found that the role of FNPs in shaping the dynamics depends on the timescales. FNPs play a crucial role in shaping rapid stimulus-evoked response dynamics <30 ms, but can be approximately neglected in favor of local geometric rules in slower time scales like resting-state fMRI settings.*

*Major Concerns:*

*While the modeling work offers some theoretical insight why some dynamical properties of macroscale cortical activity can be captured by describing the activity constrained only by the surface's two-dimensional geometry, despite neglecting the specificity of FNPs to mediate the rapid and non-local propagation of activity between remote neural populations, I have concern that the novelty of the modeling is overstated and the observed separation of the time scales is mainly stemmed from the over-simplified model without plausible biological neural dynamics, thus the interpretation of biological relevance of such over-simplified model work is likely over-extrapolated.*

**Response 1.1**

This work aims to make general claims about models that include two key phenomenological mechanisms: (i) geometric spreading as travelling waves; and (ii) rapid non-local connectomic propagation. Accordingly, we developed and analyzed a phenomenological model that captures these two mechanisms as generally as possible, as they are shared by many specifically formulated models that may also contain additional mechanisms and thus differ in their precise formulation, parameter sets, etc.

We argue that this approach is advantageous relative to analyzing a specific model formulation (that contains many more mechanisms and parameters, and would thus require justification for the many such specific choices). While some properties of simulated dynamics of more complex models would better capture some complexities found in real data, the modeling results would be less clearly generalizable. Furthermore, for any specific model formulation, the set of mechanisms and their parameters (from amongst the large number of specific choices made) that give rise to a given feature in the simulated dynamics becomes far less clear to demonstrate.

Thus, although we would expect the values reported in our paper to change with the incorporation of additional mechanisms (and associated model complexity), we would expect similar qualitative behavior from a large class of specifically formulated models that contain the two high-level mechanisms that the paper (and phenomenological model) targets.

We also note the general success of highly simplified models in capturing many macroscale neural phenomena resulting from averaging across large neuronal populations (and measured at a slow temporal resolution), in many cases outperforming more ‘biologically plausible’ models in out-of-sample testing. This includes the outperformance of linear statistical models over 'biologically plausible' models in capturing functional connectivity (e.g., Messé et al., 2015; Nozari et al., 2023), simple phenomenological models based on simple normal forms (e.g., Piccinini et al., 2022; Sip et al., 2023), and spatially isotropic geometrically constrained models (e.g., at capturing structure–function relationships despite not being constrained by structural connectivity: Pang et al., 2023). Of course models that incorporate additional complexities can more accurately account for a wider range of phenomena, but the above considerations led us to the conclusion that the phenomenological approach is most appropriate for the questions at hand, and in particular in characterizing the qualitative dynamics that emerge from a combination of geometric spreading and non-local propagation mechanisms in a model.

***Point 1.2***

*1. Novelty of the modelling*

*The author stated that they "develop a novel mathematical model of macroscale cortical activity in which cortical populations interact both by a continuous sheet and by an additional set of FNPs wired independently of the sheet's geometry". The general idea of combining local connectivity with long range projections is not new. Similar models with local geometric and long range connectome connections (in equivalent spatial discrete form) have been widely used with more realistic neural mass model enabling more realistic dynamics (e.g., Atasoy et al. Nat. Commun., 2016), including references [42-44] cited in this work. A very similar setting of the model structure with 2-d local connection and long-range connection was also studied in Liang et al, Nat. Commun. 2023) to investigate the interplay of propagating neural waves along local and long-range projections.*

**Response 1.2**

While our model formulation is, to our knowledge, novel (as stated in the quoted text), the reviewer is correct that we are not the first to develop a model containing geometric and non-local connectivity mechanisms, which was discussed in our paper (see paragraph below). We thank the reviewer for flagging an additional paper we had not cited previously; we have now incorporated it into the Discussion paragraph where we compared our formulation with prior models of this type (including contrasting their choices and limitations with ours). In addition to implementation differences (e.g., Atasoy et al. implements local connectivity as nearest neighbors on a discrete network; whereas we use a distance-dependence on a continuum, Eq. (6) [Sec. S1]), our goal is to explain the relative contribution of geometric versus non-local contributions on different timescales. We thank the reviewer for also flagging the model of Liang et al. (which is originally from Steyn-Ross et al. Phys. Rev. X, 2013) as a similar construction to our model but was not previously mentioned in our manuscript. The discussion paragraph in which we have now incorporated both prior work is reproduced below (particular edits highlighted bold):

*The results of this work were obtained from simulations of a novel mathematical model of macroscale activity which incorporates both geometric and discrete connectomic structural mechanisms within a single modeling framework (provided in open code that accompanies this work). The motivation to develop a new model is to improve upon existing models in literature that have incorporated both geometric and non-local interaction mechanisms, which can face limitations in simultaneously capturing traveling wave dynamics along a continuous sheet and rapid communication dynamics through FNPs. For example, one commonly used model is the neural mass model by Spiegler and colleagues, which simulates whole-brain stimulus-evoked responses \cite{spieglerSilicoExplorationMouse2020, spieglerSelectiveActivationRestingState2016}.*

*Our model improves upon this model by generating finite-speed traveling waves (rather than infinite-speed diffusion), and enabling simulation of dynamics in multiple contexts other than stimulus-evoked responses. Another commonly used model of this type is the neural field model by Jirsa et al. \cite{qubbajNeuralFieldDynamics2007, jirsaNeuralFieldDynamics2009, jirsaSpatiotemporalPatternFormation2000} which simulates cortical activity on a one-dimensional continuum (line) with a single embedded FNP between two points. While this model bears similarities to ours in generating traveling waves on a continuum, our model extends the one-dimensional geometry to a two-dimensional surface, to more accurately representing the geometry of the cortical sheet and enabling the model's structural connectivity to have multiple FNPs---consistent with the real cortex---without facing dynamical instabilities.*

***Also worth noting is the graph-based model introduced by Atasoy et al. \cite{atasoyHumanBrainNetworks2016} which incorporated a geometric contribution to cortical interactions via nearest-neighbor connectivity on the graph.*** *There is much scope for future work to further develop and refine models of brain activity that can simultaneously capture multiple mechanisms of cortico-cortical interactions, in order to better understand the types of interaction that shape cortical dynamics most strongly.* ***For example, simulations with the neural field model by Steyn-Ross et al. \cite{steyn-rossInteractingTuringHopfInstabilities2013}, which generalizes the model by Jirsa et al. by introducing realistic nonlinear mechanisms, suggest that introducing a bidirectional FNP can induce seizure-like bursting activity when electrical gap-junction synapses between inhibitory neurons are introduced, but otherwise minimally affects the phase coherence statistics of spontaneous dynamics.*** *Future models could also include nonlinear mechanisms to capture more realistic interactions of interfering traveling waves, such as compression and reflection in visual cortex \cite{xuCompressionReflectionVisually2007}.*

***Point 1.3***

*2. The model is oversimplified to capture biologically plausible neural dynamics.*

*(a) Model topology is over-simplified. While the model considered different settings of FNPs, the real brain connectome has far more complex architecture that has also been constrained by space across multiple scale. The claim that resting fMRI data in it's relatively slow time scale (~second) can be well captured by local connections, but ignoring FNP is not well justified. There is an oversimplified assumption made in Eq. (32), i.e. that the BOLD signal can be approximated by the time-integral of ϕ(r,t) (by retaining only the ω=0 Fourier component). Neuro-vascular coupling is not pure direct mapping. In previous experimental and modelling works, the BOLD response is generated by convolving neuronal activity with HRF or through the Balloon-Windkessel model. Consequently, the BOLD signal preserves a range of low-frequency components. It's not clear and cannot be inferred from the current work how the multilevel, spatially constrained connectome and the multiscale temporal dynamics could induce much broader spatiotemporal effects of the FNP, different from the findings of spatially and especially temporally short-range activation on top of spontaneous activity here. While previous work [28, 29] showed that the idealized geometric connectivity rule of neural field models can provide valid approximation for FMRI patterns, it cannot rule out the contributions from the strongly spatial-constrained components of the long-range connectome, especially on temporal dynamical features (not just spatial functional connectivity ignoring temporal dynamics). Indeed, a huge body of work on structure-function relationship has shown that the structural connectome can reasonable explain the functional interaction of resting fMRI.*

**Response 1.3**

See Response 1.1 above for our general motivation for the justification for our phenomenological approach for capturing the core qualitative mechanisms as generally as possible. To the specific points:

(i) We have indeed simplified the topology of FNP topology, focusing on providing a clear understanding of how each FNP can be viewed as a perturbation to geometric dynamics. To give a sense of how these results may play out in the presence of a network of perturbatory FNPs, we investigated the contribution of simple non-trivial topologies (including hub and rich-club connectivity) in the section titled “Influence of a complex network of FNPs on cortical dynamics”.

(ii) We agree that our BOLD model is a vast simplification. Taking the zero-frequency limit is a useful simplification with precedent (e.g., Robinson et al., Neuroimage (2006)) and is sufficient for addressing the questions we use it to tackle in this work. In the revision, we have improved our discussion of this limitation in the paper, including more explicitly describing alternative choices that could be made to better capture BOLD dynamics on shorter timescales:

*At each $\mathbf{r}$, the BOLD response was approximated as $z(\mathbf{r})$, the stimulus-evoked response's zero temporal frequency component~\cite{robinsonDeterminationEffectiveBrain2014}, or equivalently, the time-integrated response as depicted schematically in Fig.~\ref{fig:fMRI}A (see Eq.~\eqref{SI:eq:bold} in \nameref{SI:NumericalTreatment} for details on how $z(\mathbf{r})$ is computed numerically). This linear approximation closely aligns with, and hence can be generalized to BOLD responses computed with more complex nonlinear hemodynamic forward models~\cite{robinsonBOLDResponsesStimuli2006}* ***that preserve more low-frequency dynamics,*** ***including physiologically realistic \cite{*Aquino2014:DeconvolutionNeuralDynamics*} and phenomenological \cite{Friston2000:NonlinearResponsesFMRI} constructions.***

(iii) We agree with the reviewer’s remaining points that, although neural field theory has been surprisingly successful in capturing many properties of fMRI dynamics using highly idealized model constructions, it is a simplified model with substantial limitations, and many dynamical properties of neural activity are well-established to be constrained by structural connections and their topology in the connectome. Indeed, our results demonstrate clearly that ‘smearing the specificity’ of structural connectivity into an isotropic geometric approximation is a poor approximation on fast timescales, but becomes a better approximation on increasingly slower timescales (and under more spatially homogeneous drives). Our work does not aim to make a case that diminishes the contribution of specific long-range connectomics to cortical dynamics but rather, by providing an account of the timescales on which different mechanisms may play a dominant role in shaping neural dynamics, our work attempts to provide constraints on what we can expect to see under such circumstances. We hope the resulting predictions could provide opportunities for experimental validation in future work. This framing, which captures the strengths of modeling structural connectivity directly (and the approximation of using simply its geometric ‘smearing’) is outlined in the opening paragraph of the Discussion:

*These empirical results, across scales and species, demonstrate the important role of FNP networks in facilitating the complex, spatially distributed cortico-cortical communication thought to underpin the brain's remarkable cognitive functionality, despite their costs to develop, maintain and operate \cite{vandenheuvelHighcostHighcapacityBackbone2012, vertesGeneTranscriptionProfiles2016, fulcherTranscriptionalSignatureHub2016, arnatkeviciuteGeneticInfluencesHub2021}. On the other hand, a second collection of results demonstrates the success of neural field models which, via a spatially homogeneous and isotropic connectivity rule assumption \textit{neglect} specific FNPs, but can nevertheless predict many aspects of cortical dynamics accurately, particularly the spontaneous fluctuations observed on the order-of-seconds timescales of fMRI \cite{robinsonDeterminationDynamicBrain2021, hendersonEmpiricalEstimationEigenmodes2022, pangGeometricConstraintsHuman2023}. This work sought to address the question: how can models of cortical dynamics that `smear out' the anatomical specificity of FNPs to a geometric average nevertheless generate such accurate predictions of cortical dynamics?*

***Point 1.4***

*(b) Model lacks of biologically plausible dynamics. The model derivation focused on the physical aspects of propagation of activity across homogeneous 2-D space (real brain regions also show regional heterogeneity/hierarchy which is also coupled, but not independent with the long-range connectome) , around a steady state in a linear manner. Thus, the key equations describe passive response from external background noise or target stimulation largely through passive diffusion (damped wave equation), and activity is fully driven by external signal, without internal self-oranization. It's well documented that neural dynamics is highly nonlinear and far from equilibriums, characterised by scale-free features in space and time and signature of criticality. In such system, local activation can scale-up with spatial long correlation length. Such "active'' matter can also act very differently under different states (e.g., anaesthesia or wakefulness), and the spatiotemporal patterns are constrained, but not completely slaved by the underlaying structural topology (which unfortunately, is the main property of the model here). The structure-function coupling depends on brain states. Thus, the observed well separation of fast and slow time scale presented here may not be plausible for biological neural dynamics. For example, Liang et al 2023 showed that long-range projection on the 2-D space may or may not propagate signal to create new wave sources depending on the excitability of the circuit.*

**Response 1.4**

We agree that models exhibiting nonlinear, far-from-equilibrium dynamics are crucial to accurately capture the nonlinear dynamics of some complex neural phenomena. Here we model spontaneous dynamics, which have been shown to be well-captured at the macroscale by models operating under an approximately linear ‘noisy fixed-point’ regime (Sip et al., 2023), or in some cases near a Hopf bifurcation (Ponce-Alvarez & Deco, 2024), as per the parameters used in many neural field and neural mass models of resting-state fMRI data. This includes many biophysically plausible models that include nonlinearities in principle, but which in practice often operate in such near-linear regimes when modeling spontaneous dynamics. Our core argument for model simplifications of this type is similar to that made in responses above: modeling specific forms of nonlinearities would complicate the generalizability of the model in capturing phenomena distinct from the two key mechanisms that were the target of this work.

We agree that spatiotemporal dynamics are importantly constrained by fluctuations in properties like cortical excitability (e.g., from neuromodulation), that were not included in our simplified model. We have now expanded our discussion of this point in the Discussion:

*A notable limitation of this work is that our modeling results, and their interpretation, rest on specific assumptions made in constructing the model.*

*[...]*

***Our model also assumes a system gain or excitability (model parameter $\nu_0$) that is constant over time, however it is known that the brain can operate at different levels of excitability over time, regulated by neuromodulatory systems of the subcortical brainstem and forebrain \cite{munnAscendingArousalSystem2021, orlandoNoradrenergicCholinergicSystems2023}. A sensitivity analysis in \nameref{SI:fig:sensitivity}A shows that the FNP's perturbation on stimulus-evoked dynamics when the stimulus is at the source of FNP tends to persist more over long timescales as gain reduces. Furthermore, the perturbations that FNPs induce on long-timescale spontaneous dynamics have been shown to amplify when the dynamics operates at higher excitabilities \cite{robinsonNearcriticalCorticothalamicEigenmodes2025, liangComplexityCorticalWave2023}. Future work could investigate how neuromodulatory systems could influence FNP-induced perturbations on both spontaneous and stimulus-evoked cortical dynamics.***

***Point 1.5***

*Below there are also a few other more concrete points which are related to biologically unrealistic over-simplifications largely outlined above.*

*(1) In the Model Implementation, whether it is reasonable to set all τ_m=0 depends on the functional role ascribed to the FNPs and on the temporal scales relevant to the questions being addressed. The domain is a square with L=0.4 m, topologically wrapped in a torus. Given the wide range of typical cortical axonal conduction velocities, the corresponding propagation times would therefore be varied. The times are clearly not negligible compared with synaptic and neuronal time constants (up to 30 ms) and with many cortical rhythms. Consequently, setting τ_m=0 can only be regarded as a first-order simplification if the FNPs are interpreted as instantaneous, field-like couplings, or if the phenomena of interest unfold on time scales much longer than 50 ms. For precise modelling of long-range feedback, the delays should be restored.*

**Response 1.5**

We agree: setting *τ_m = 0,* corresponding to instantaneous conduction, is a major simplifying approximation used in this work. We note that this assumption is commonly made in the literature, and in this work it allowed us to qualitatively isolate the ‘fast’ nonlocal dynamics from the slower geometric propagation, as is the focus of our investigations, expecting similar dynamics in the limit of the resulting timescale separation. We highlight this limitation in the Discussion:

*We also made the major simplifying assumption of modeling FNPs in the limit of instantaneous conduction, $\tau_m = 0$, which neglects time delays due to physical conduction. Although an idealization, this choice allowed us to more straightforwardly model and qualitatively understand the qualitative effects of rapid non-local projections that arrive at some distant target location much faster than a traveling wave. Human diffusion MRI data reveals that the ratio between a structural fiber's geodesic length (along the cortical surface) and three-dimensional length through the white matter is approximately $7$ for the fibers with the largest geodesic lengths, but approximately $2.5$ for the average connection \cite{hendersonRelationsGeometryCortical2014}. Since the axonal conduction velocity in neural field models, $v = r \gamma$, is parameterized to incorporate this ratio, a reasonable choice for the time delay of a given FNP $\mathbf{a}_m\to\mathbf{b}_m$ is $\tau_m = d_m/v \times (2.5/7)$, where $d_m = \|\mathbf{a}_m - \mathbf{b}_m\|$ is the geodesic length of the FNP.*

***Point 1.6***

*(2) In the hybrid model, FNP is set to propagate perturbations from one point to another without any amplification or attenuation (with a constant c_m=r^2), which is an idealized assumption, as more plausible dynamics is not modeled in the system. In the Discussion section, the authors have mentioned that "…however the perturbations that FNPs induce on long-timescale linear cortical dynamics can be shown to be amplified when the dynamics operates closer to its critical point [85]". In the main text it still lacks relevant experiments or systematic statement to clarify if the main conclusions remain robust when more realistic neural dynamics is considered.*

**Response 1.6**

We agree that our choice of a fixed long-range connectivity strength *c_m* is a simplifying assumption. While the perturbation of the FNP on the dynamics is expected to increase with its connectivity strength, we did not expect the qualitative dynamics of the perturbation to change. To verify this expectation, we have recalculated the perturbation curve *C(t)* (using nominal parameters in Figure 3B) for a range of values of connectivity strength to the supporting information, as Fig. S4B (reproduced below).

The new result is now referenced in the updated Discussion:

*Other simplifying assumptions include setting the connectivity strength of each FNP, $c_m$, to a fixed value of $r^2$.* ***While the overall behavior of the perturbation of an FNP over time is not expected to change with different values of $c_m$ (see Fig. S4B for the dynamical perturbation of a single FNP for different values of $c_m$), future work could better calibrate this value (e.g., using structural connectivity measurements) to yield more accurate simulations in specific settings.***

*[...]*

***Point 1.7:***

*(3) The manuscript states that the value of υ_0 is adopted directly from the excitatory-only neural field model developed by Jirsa and Haken. This is the only parameter describing the local dynamics. Because the present conclusions may depend on the specific numerical choice made for υ_0. It is necessary to: (a) Conduct a sensitivity analysis to show that the reported phenomena are robust to the variation of υ_0. (b) Clarify whether υ_0 is fixed for all simulations or allowed to change with other parameters (e.g., synaptic strength, etc.).*

**Response 1.7**

First, we confirm that nu_0 is fixed through all simulations. We have now clarified this:

***The value of $\nu_0$, which is the effective rate at which activity at a given position is regenerated, is fixed in all experiments conducted here, and was taken from the excitatory-only neural field model developed by Jirsa and Haken \cite{jirsaDerivationMacroscopicField1997}.***

We did not expect the qualitative dynamics—which our paper aims to capture—to be highly sensitive to our choice of nu_0. When we decrease nu_0, we expect *C(t)* to persist over longer timescales, because our investigations require the traveling wave to be sufficiently sustained as they traverse the surface. We have now conducted a sensitivity analysis to verify this (Fig. S4A, reproduced in Response 1.6 above) and have made reference to the result in the updated Discussion:

*A notable limitation of this work is that our modeling results, and their interpretation, rest on specific assumptions made in constructing the model. [...]*

***Our model also assumes a system gain or excitability (model parameter $\nu_0$) that is constant over time, however it is known that the brain can operate at different levels of excitability over time, regulated by neuromodulatory systems of the subcortical brainstem and forebrain \cite{munnAscendingArousalSystem2021, orlandoNoradrenergicCholinergicSystems2023}. A sensitivity analysis in Fig. S4A shows that the FNP's perturbation on stimulus-evoked dynamics when the stimulus is at the source of FNP tends to persist more over long timescales as gain reduces. Furthermore, the perturbations that FNPs induce on long-timescale spontaneous dynamics are shown to amplify when the dynamics operates at higher excitabilities \cite{robinsonNearcriticalCorticothalamicEigenmodes2025, liangComplexityCorticalWave2023}. Future work could investigate how neuromodulatory systems could influence FNP-induced perturbations on both spontaneous and stimulus-evoked cortical dynamics.***

***Point 1.8***

*Overall, this work is a more physics-based modelling and can provide some theoretical insight. However, the main results may be specific to the oversimplified model. Without a more realistic brain network topology and most importantly plausible neural dynamics calibrated by multi-modality data (e.g., EEG vs fMRI), it's limited novelty and unclear biological relevance make it unsuitable for publication in PLoS Biology. The work presented here perhaps fits better for physics journals (e.g. Physical Review E). It could also be suitable for PLoS Computational Biology if the claims could be tuned down properly in the light of strong simplification of the modelling, especially on neural dynamics.*

**Response 1.8**

We thank the reviewer for the thoughtful and detailed comments that have allowed us to improve the manuscript. We hope that the above responses help to clarify our goals and the suitability of a phenomenological model in addressing them, our improved interpretation and framing of the results, and note our resubmission of this manuscript to *PLoS Computational Biology*, whose audience is better suited to the paper’s contributions.

# **Reviewer #2:**

***Comment 2.1***

*This manuscript develops a novel theoretical and computational framework that unifies geometry-constrained neural dynamics with long-range projections in a neural field setting. Specifically, it extends neural field theory by formulating a non-local partial differential equation (PDE) with activity-preserving properties, solved numerically on a torus geometry using finite-difference methods. The authors systematically explore stimulus-evoked and noise-driven regimes across various connectome topologies, quantifying the influence of fast non-local projections (FNPs) through measures such as cosine dissimilarity.*

*The work is, for the most part, well written, mathematically rigorous, and clearly presented. Its main theoretical contribution lies in the mathematical and computational integration of geometry-driven dynamics with long-range projections. Its main neuroscientific contribution is to provide a mechanistic account of how structural constraints differentially influence cortical dynamics across timescales: the simulations convincingly show that FNPs strongly perturb rapid dynamics (<30 ms) but have limited impact on slower spontaneous fluctuations (>30 ms). This offers a plausible explanation for divergent empirical findings (EEG/VSD vs. fMRI) and constitutes a timely contribution to current debates on geometry-connectome interactions in shaping brain dynamics.*

*While the manuscript is strong, several conceptual and methodological aspects should be clarified or expanded, as detailed below.*

**Response 2.1**

We thank the reviewer for their thoughtful reading and assessment of our work which has resulted in a range of improvements in this resubmission to *PLoS Computational Biology*.

***Comment 2.2***

*Major Comments*

*1. Interpretation of FNP influence on slow dynamics: The claim that "While FNPs specified by the connectome are needed to capture rapid communication between specific distant populations (as per the rapid processing of sensory inputs), they play a relatively minor role in shaping slower spontaneous fluctuations (as per resting-state functional magnetic resonance imaging)" appears to be directly challenged by Vohryzek et al. (2025, PNAS, Ref. 81). This study shows that rare long-range structural connections play a key role in shaping resting-state dynamics, significantly improving model fits to functional connectivity and time-lag structures compared to geometry-only models. The manuscript cites this work but does not acknowledge or resolve this contradiction. This point should be explicitly discussed in the Discussion.*

**Response 2.2**

We first note the context of the reference to the work of Vohryzek et al. made in the Discussion, e.g.,:

*[...]*

*Note that this does not imply that the specificity of the connectome is unimportant for capturing resting-state fMRI dynamics---indeed, the further constraining of geometric connectivity with specific connections from connectomic data can improve predictions of resting-state fMRI dynamics \cite{decoRareLongrangeCortical2021, vohryzekHumanBrainDynamics2025, decoNonlocalSchrodingerDiffusion2025}.*

*[...]*

We clearly acknowledged in our original paper (and supported by our own simulations) that fitting more complex models that include long-range connectomic information (i.e., EDR+LR) can lead to an improvement in fitted accuracy. And in the text quoted by the reviewer we claim that the role is “relatively minor” for “shaping slower spontaneous fluctuations”. These claims are consistent with the results of Vohryzek et al. (2025) where, in attempting to reconstruct fMRI maps, they demonstrated a small but significant degree of improvement when using long-range connectivity information (‘EDR+LR’) relative to isotropic geometric connectivity (‘EDR continuous’).

We have reproduced Figs 2B,C from Vohryzek et al. (2025) below; we can see the benefit in reconstruction accuracy (EDR continuous [orange] -> EDR+LR [purple]) in panel C is down to the third decimal point in reconstruction distance and barely perceptible visually, even after zooming in on the position of the largest such difference in favor of EDR+LR (at 200 modes; whereas EDR+LR has poorer performance at lower mode numbers).

The finding of Vohryzek et al. (2025)—that there is a relatively small gain in accuracy from incorporating long-range connections in capturing slow spontaneous brain dynamics—is in agreement with our results, which support the role of long-range connections in modulating brain dynamics particularly on fast timescales, with a relatively lesser contribution on the longer timescales of fMRI.

***Comment 2.3***

*2. Non-local term formulation (Lines 83-95): One of the manuscript's most important theoretical contributions is the introduction of the non-local term $C \circ \phi$ in the differential equation. However, its formulation is described only briefly, and the Methods section does not clearly explain how connectivity enters the equation. The Supporting Information provides equations but does not specify how parameters such as $c_m$ and $\tau_m$ are chosen to link the model to the underlying connectivity. This key information should appear explicitly in the Methods, rather than being deferred to the SI.*

**Response 2.3**: We agree that this detail is better explained clearly in the main text. The Methods section contains information about how c_m and tau_m are specified:

**The formulation for the non-local term $(C \circ \phi)$ is given by**

**\begin{align}**

**(C \circ \phi)(\mathbf{r}, t) = \sum_m c_m \big[ \delta(\mathbf{r} - \mathbf{b}_m) \phi(\mathbf{a}_m, t - \tau_m) - \delta(\mathbf{r} - \mathbf{a}_m) \phi(\mathbf{a}_m, t) \big].**

**\end{align}**

**As detailed in \nameref{SI:modelderivation}, $\mathbf{a}_m, \mathbf{b}_m$ are positions of the source and target populations of the $m^\text{th}$ FNP, and $c_m$ physiologically represents the effective increase in activity at the target population per unit activity that the FNP exhibits at its source.**

We set $c_m$, which is the connectivity strength of the $m$th FNP, to equate the strength of local and non-local propagation from the source of the FNP. Accordingly, we set $c_m = r^2$ for each $m$, so that when the model is numerically treated, the connectivity strength of the FNP ($c_m/(\Delta x)^2$ in Eq.~\eqref{SI:eq:nonlocalnumericaltreatment} in \nameref{SI:NumericalTreatment}) is equal to the strength of coupling between geometrically nearest neighbors on the grid from the local geometry ($r^2/(\Delta x)^2$ in Eq.~\eqref{SI:eq:laplaciannumericaltreatment} in \nameref{SI:NumericalTreatment}). We set the value of $\tau_m$, which represents the conduction time of the $m$th FNP, so that it is always smaller than the conduction time of a traveling wave between the FNP source and target, but is also small enough to avoid oscillatory instabilities independently of the chosen connectome configuration \cite{qubbajNeuralFieldDynamics2007, jirsaNeuralFieldDynamics2009}. However, the conduction speeds of FNPs vary widely from factors other than their length \cite{swadlowAxonalConductionDelays2012}, and the critical values of the $\tau_m$ values separating stability from instability depends on the spatial configurations of the FNPs \cite{qubbajNeuralFieldDynamics2007}. For this reason, we fixed $\tau_m = 0$ for each $m$.

***Comment 2.4***

*3. Geometric domain choice: The use of a torus is understandable from a numerical perspective, but the biological justification is weak. Neural field theory has historically considered spherical topologies. Why was this alternative not tested here? Are there arguments suggesting that different boundary conditions (e.g., sphere vs. torus) would not affect the conclusions?*

**Response 2.4**

We note that neural field theories have most commonly used a toroidal geometry, as periodic boundary conditions on a square or rectangular domain (e.g., Robinson et al. 1997; Robinson 2003; Sanz-Leon et al., 2018), as we use here. This choice is common, in part because it allows for a straightforward spatial Fourier decomposition in terms of plane wave eigenfunctions/modes (which is much more complex for a spherical geometry).

We would expect similar qualitative dynamics for another closed surface like a sphere, because e.g., the qualitative behavior of *C*(*t*) in Fig. 3B (increasing and then decreasing back to zero) is caused by the traveling wave from *r*_1_ and *r*_2_ reaching all points on the surface [as explained in the Results subsection “Influence of a single FNP on cortical dynamics resolved across varying timescales”]. We have added a clarification of this specific point to the updated Discussion (when discussing potential extensions of the work to other geometries):

*[...]*

***Note that our experiments are set up such that the qualitative results are not highly sensitive to the periodic boundary conditions and we expect similar results on other closed surfaces such as spherical domains.***

*[...]*

***Comment 2.5***

*4. Network models and empirical data: The network models are not fully described in the Methods, despite being central to the study. The abstract states "By simulating the model across a range of connectome topologies," and five pages of results depend on these models. Including a clear description of the random connectivity model and its three parameters in the Methods would improve readability. Moreover, these models rely on three basic structural constraints (exponential distance, hub specificity, rich-club specificity), rather than empirical connectomic data. Real connectomes display a broader set of local and global properties (e.g., heavy-tailed degree distributions, hierarchical modularity, low effective rank). It would strengthen the manuscript to discuss how these simplifications might affect the conclusions.*

**Response 2.5**

We thank the reviewer for the comment and agree that additional clarification is required here.

We have rephrased the sentence quoted from the abstract to hopefully give a more accurate impression that the topological investigation is not central to the study, but included to demonstrate that the core insights of considering individual connections generalize to the setting of multiple connections:

“By simulating the model across a range of external inputs, timescales, **and idealized connectome topologies**, [...]”

We have also followed the reviewer’s suggestion and provided clearer context for how the connectome experiments fit within the scope of the paper as a new paragraph added to the *Methods* section:

***The majority of this paper characterizes the effect of a single FNP as a perturbation to a background of geometrically constrained connectivity, in shaping the response to an impulsive stimulus of a given spatial profile and for spontaneous dynamics. We then investigate the results of a network of FNPs embedded in the domain $\Omega$, considering random (unconstrained) connectivity as well as a range of idealized constraints on connectome wiring inspired by network structures observed in real structural connectome data: (i) distance-dependent wiring of FNPs via an exponential distance rule (indexed by a given lengthscale $\lambda_e$) \cite{ercsey-ravaszPredictiveNetworkModel2013}; (ii) a hub-specificity constraint (that preferences connections to involve specified hub locations, indexed by parameter $\lambda_h$) \cite{markovRoleLongrangeConnections2013}; and (i) rich-club specificity (that preferences hub--hub connectivity, indexed by parameter $\lambda_r$) \cite{vandenheuvelHighcostHighcapacityBackbone2012}. Details of each algorithm for generating a given arrangement of FNPs are described in \nameref{SI:ResultsDetails}.***

And finally, we have deepened our discussion on how the highly idealized topological simplifications may affect the conclusions in the updated Discussion:

*[...]*

*Finally, the cortical surface $\Omega$ was set to have a toroidal geometry, and nonrandom FNPs on $\Omega$ were sampled algorithmically under idealized topological constraints, rather than from connectomic data.* ***This simplification ignores other aspects of connectomes, such as modularity and heavy-tailed degree distributions \cite{spornsModularBrainNetworks2016}, which may affect the conclusions reached in our investigations.*** *Future work could thus investigate the effects of instead modeling the real geometry $\Omega$ of a cerebral hemisphere \cite{gabayCorticalGeometryDeterminant2017} and/or aligning the wiring configurations of all FNPs with connectomic data as pursued in previous work \cite{decoRareLongrangeCortical2021, vohryzekHumanBrainDynamics2025}.* ***Such work would involve more complex computation of the Laplace-Beltrami operator ($\nabla^2$), as it must incorporate the irregular sampling of points, and varying point-to-point distances that are manifested in convoluted cortical mesh data \cite{pangGeometricConstraintsHuman2023, gabayCorticalGeometryDeterminant2017, hendersonEmpiricalEstimationEigenmodes2022, robinsonDeterminationDynamicBrain2021}.***

*[...]*

***Comment 2.6***

*5. It would also be valuable for the authors to discuss how their framework could be applied to empirical data, such as realistic human cortical geometries or connectomic datasets, or to clarify whether there are strong conceptual or technical reasons that justify the simplified geometry and connectivity used here.*

**Response 2.6**: We agree. As the reviewer notes, we chose to focus here on simplified geometries and connectomic datasets to allow for explanatory/mechanistic understanding in a general phenomenological model containing the core mechanisms at play. Our Discussion now contains a sentence on how the model could be used to simulate real data in future work:

*[…]*

*Future work could thus investigate the effects of instead modeling the real geometry $\Omega$ of a cerebral hemisphere \cite{gabayCorticalGeometryDeterminant2017} and/or aligning the wiring configurations of all FNPs with connectomic data as pursued in previous work \cite{decoRareLongrangeCortical2021, vohryzekHumanBrainDynamics2025}.*

***Such work would involve more complex computation of the Laplace-Beltrami operator ($\nabla^2$), as it must incorporate the irregular sampling of points, and varying point-to-point distances that are manifested in convoluted cortical mesh data \cite{pangGeometricConstraintsHuman2023, gabayCorticalGeometryDeterminant2017, hendersonEmpiricalEstimationEigenmodes2022, robinsonDeterminationDynamicBrain2021}.***

***[****…]*

***Comment 2.7***

*6. Dimensionality and 3D extension: A central motivation for the manuscript is the geometric-functional relationship recently studied by Pang et al. While that work has received criticism, one underexplored aspect concerns subcortical and volumetric (3D) regions. Recent computational and experimental work (e.g., zebrafish functional imaging) has examined this relationship in three-dimensional volumes. In contrast, the present manuscript is restricted to 2D surfaces, which represents an important limitation. This should be explicitly acknowledged, along with a discussion of the feasibility of extending the framework to 3D.*

*Do the authors intend or expect their theoretical formulation to extend naturally to three-dimensional domains? If not, what are the main conceptual or technical obstacles?*

**Response 2.7**

We thank the reviewer for raising this interesting point. We agree that it would be an interesting and fruitful direction of future research to extend the approach to 3D. We have now added some text on this point to the updated Discussion:

*[…]*

*Future work could thus investigate the effects of instead modeling the real geometry $\Omega$ of a cerebral hemisphere \cite{gabayCorticalGeometryDeterminant2017} and/or aligning the wiring configurations of all FNPs with connectomic data as pursued in previous work \cite{decoRareLongrangeCortical2021, vohryzekHumanBrainDynamics2025}.* ***There is also scope to extend this work to three-dimensional domains, as was explored in a recent investigation with a nearest-neighbor neural mass model (different to a wave equation on a continuous surface) \cite{legareFunctionAlignsGeometry2025}, however we remark that the connectivity rule that is encoded in the three-dimensional damped wave equation, while still isotropic, lacks the decaying property that is manifested in the connectivity rule encoded in the two-dimensional equation \cite{robinsonPropagationStabilityWaves1997}.***

***Comment 2.8***

*Minor Comments*

*1. Introductory claims: The introduction states that "many dynamical properties of macroscale cortical activity" can be explained by geometry-only models. It would help to specify which properties are meant and provide explicit examples to clarify the scope of the claim.*

**Response 2.8**:

We have now provided additional detail on this point:

*Despite extensive evidence demonstrating the key role of FNPs in supporting cortical function and shaping cortical dynamics, recent analyses have indicated that many dynamical properties of macroscale activity (on millimeter lengthscales and above),* ***such as resting-state functional magnetic resonance imaging (fMRI), the spatial patterns of fMRI task activation maps, and resting-state magnetoencephalography (MEG)****, can be well captured by geometrically constrained models of neural activity that neglect the specificity of connectome-informed FNP connectivity \cite{robinsonDeterminationDynamicBrain2021, hendersonEmpiricalEstimationEigenmodes2022, pangGeometricConstraintsHuman2023}.*

***Comment 2.9***

*2. Connectomic literature: Line 17: "several high-profile experimental efforts have aimed to develop comprehensive connectomes across species." The list provided is not exhaustive. For a broader overview, see Barabási, Dániel L., et al. (2023, J. Neurosci.) and Lin, Albert, et al. (2024, Nature).*

**Response 2.9**

We have now incorporated these additional references:

[...]

*Accordingly, several high-profile experimental efforts have aimed to develop comprehensive connectomes across species, which detail the specific populations that FNPs (as well as other fibers) connect \cite{ohMesoscaleConnectomeMouse2014, vanessenWUMinnHumanConnectome2013, kotterOnlineRetrievalProcessing2004, linNetworkStatisticsWholebrain2024, barabasiNeuroscienceNeedsNetwork2023}, and hence significantly accelerate our understanding of the structural underpinnings of cortical function and dysfunction \cite{bargmannConnectomeBrainFunction2013, fornitoConnectomicsNewParadigm2015}.*

***Comment 2.10***

*3. Terminology: the manuscript's use of "hub" differs from standard network neuroscience usage. In the field, a hub is a highly connected node, whereas here it refers to a spatial module or community. Similarly, what is described as a "rich club connection" resembles a stochastic block model. Adopting standard terminology would improve clarity. See Bassett et al. (2018, Nat. Rev. Neurosci.) and Faskowitz et al. (2018, Sci. Rep.).*

**Response 2.10**

We thank the reviewer for flagging this source of confusion in our presentation of hubs. Because we are resolving the cortex on a continuous spatial domain, we required a definition of a hub in this domain: as an *area* with preferential connectivity; after parcellation of the space, the node corresponding to this cortical area would be a hub. This is now clarified in the revised manuscript:

*Hub specificity captures the empirical observation that specific hubs of the cortex tend to be particularly strongly connected and are thought to play a major role in mediating distributed information transfer across the cortex \cite{markovRoleLongrangeConnections2013, betzelSpecificityRobustnessLongdistance2018, betzelGenerativeModelsHuman2016}.*

*[…]*

*At $\lambda_h = 0$ we see spatially uniform random FNP connectivity unaffected by the assignment of hub areas, whereas at $\lambda_h = 1$ we see that all FNPs connect with a hub.*

***We remark that, given our continuous spatial formulation, a hub here is treated as a spatial area of the cortical sheet with preferential connectivity; if this continuum were to be parcellated into discrete nodes, the node(s) corresponding to such high-connectivity areas would become highly connected hub nodes in the resulting network (as would be analyzed in network neuroscience).***

***Comment 2.11***

*4. Table 1: The length parameter $L$ is listed under "Topology," but $L$ sets the metric scale, not the topology. The torus topology arises from the periodic boundary conditions, not from the value of $L$. Consider clarifying this in the table caption to avoid confusion.*

**Response 2.11**:

We agree that this terminology could be confusing. We have now renamed ‘topology’ to ‘medium’.

# Reviewer #3:

***Comment 3.1***

*In this submission the authors address questions about signal propagation in brain networks. They model two competing pathways for propagation - local geometry and long-distance projections. This is an important topic and one that has ignited some debate in the last few years (see Pang et al 2023 Nature, which made the argument that local geometry is sufficient to explain many phenomena, which did not sit well with others; for instance, see Faskowitz et al 2023 bioRxiv). In this way, the results shared here are diplomatic—they posit that geometry and long-distance connectivity both play important roles, albeit in a context-dependent way: over short timescales the long connections make a big impact, whereas this is not the case of longer timescales.*

*Overall, I think it's a very nice paper. But I'm not sure that it is the major conceptual or biological advance that the authors claim. It strikes me as not particularly novel—there have been other papers examining the effect of long-distance connections on dynamics (DOI: 10.1038/ncomms8723 and* [*https://url.au.m.mimecastprotect.com/s/sykWCZY1Nqi8Oq2Y0IjhlCB_UjN?domain=doi.org*](https://url.au.m.mimecastprotect.com/s/sykWCZY1Nqi8Oq2Y0IjhlCB_UjN?domain=doi.org)*). I also think it is a big missed opportunity to include a stronger empirical validation of the model (though I also see the advantage of testing the model on parameterized synthetic, toy networks).*

*So: Good, solid paper. A little light on biology. Would be great for PLOS Comp Biol.*

**Response 3.1**:

We thank the reviewer for their time to read and thoughtfully assess our work and its importance in the context of the field.

Regarding the level of advance, we do not claim to be the first to study the dynamics of spatially embedded phenomena with long-range perturbations (and we discuss much such prior work in our manuscript); our core contribution is rather to characterize how the geometric spreading versus long-range interaction mechanisms shape the dynamics over distinct timescales, which provides a potential reconciliation of the recent debate characterized by the reviewer. In particular, why geometric smearing of the connectome is a better approximation on long timescales, whereas connection specificity is very important on shorter timescales. To our knowledge, prior work has not demonstrated this phenomenon, and has not provided such a reconciliation of this major open problem in neuroscience. To briefly distinguish our work from the two cited works:

Taylor et al. (*Nat. Comms.,* 2015) examine contagion/epidemic phenomena unfolding on spatially embedded networks using topological data analysis. We thank the reviewer for drawing our attention to this paper, which we were not aware of. It is related to our work insofar as they combine spatial embedding with long-range shortcuts, but it does not address neural phenomena, nor does it address the separation of timescales on which long-range perturbations substantially shape dynamics that is core to addressing the question posed in our work.

Vohryzek et al. (PNAS, 2025) compare eigenmode-based reconstruction of fMRI maps using an isotropic exponential distance rule (EDR) to that obtained by also incorporating long-range connections (as was done in Pang et al. (2022)). At a large number of modes (200), they demonstrate a statistically significant but minimal benefit to reconstruction accuracy when incorporating information long-range connections over the EDR model (cf. Fig. 2B,C from this paper reproduced below: comparing EDR continuous to EDR+LR in panel C). This small improvement is consistent with our results that would predict a minimal benefit to incorporating long-range connections on the long timescales of fMRI. Importantly, this paper *does not treat dynamics at all* (such that it cannot make claims about timescales of response to perturbations).

In summary, to our knowledge, no prior work has provided a compelling explanation for the dynamical role of long-range connections in playing different roles on different timescales, as developed by us in this work. We note that the two papers above are incorporated into our manuscript as below, firstly:

*[...]*

*Note that this does not imply that the specificity of the connectome is unimportant for capturing resting-state fMRI dynamics---indeed, the further constraining of geometric connectivity with specific connections from connectomic data can improve predictions of resting-state fMRI dynamics \cite{decoRareLongrangeCortical2021, vohryzekHumanBrainDynamics2025, decoNonlocalSchrodingerDiffusion2025}.*

*[...]*

And the Taylor et al. paper has now been incorporated into the Discussion:

*[...]*

*We also note the potential of interdisciplinary connections between our findings and other spatially embedded phenomena with long-range perturbations, such as work modeling social or biological contagion as a dynamical process evolving on a spatially embedded network with additional rapid non-local interactions (e.g., from international air travel or internet-mediated social interactions) \cite{Taylor2015:TopologicalDataAnalysis}.*

***Comment 3.2***

*Major comments*

*1. The authors set up the problem as: "what is the point of long-distance connections if surface geometry does so well?" This isn't quite right, I think. One reason surface geometry does well is likely due to the rarity of genuine long-distance projections. They appear infrequently empirically but also in models, e.g. exponential distance rule. The dominant architectural feature in connectomes is short-range.*

**Response 3.1**

We note that the formulation of most neural field theories are consistent with a connectivity strength that decays exponentially with distance; the geometric form of our model is of this type, whereby long-range projections are rare (exponentially as $exp(-\lambda*d)$). Our model includes rapid ‘shortcut’ perturbations to this background ‘isotropic smearing out’ of average connectivity, in which specific rapid long-range connections can indeed dominate cortical dynamics on slow timescales. This is most salient in say a whisker-stimulation experiment where a sensory input leads to rapid activation of a distant neural population connected via a strong axonal pathway (eventually the geometric connectivity ‘washes out’ these highly non-local short-timescale dynamics). The key contribution of our work is to emphasize how important these very specific (and often genetically coded) long-range projections are for rapid and distributed neural processing, while also proposing a plausible account for why it is often a reasonable approximation to average over them as an isotropic exponential distance rule on longer timescales.

***Comment 3.3***

*2. Relatedly, I worry that the authors are putting too much stock in the Pang et al paper. Though it quite convincingly demonstrated that surfaces are great for explaining features of BOLD fMRI, there are myriad other imaging/recording modalities and it remains unclear if the geometric mode-based approach will be as successful there as it was with BOLD fMRI. The same issue holds for spatial scale - geometric modes work well at meso/macroscale where we model our data as continuous surfaces, but would it work equally well at single neuron resolution where our neural elements are fundamentally discrete? I'm not sure. For this and the previous point, I would ask for some balance in discussion. Pang et al is interesting and has reignited debate; it has not closed the book on explaining the origins of brain function/dynamics.*

**Response 3.3**

The reviewer seems to be characterizing the simplified argument that ‘geometry is all you need’. We agree with the reviewer in completely rejecting this un-nuanced framing, and we took some care to avoid this implication in our work, as it runs counter to our presentation of the problem and our main results.

Our framing points to clear evidence for which the geometric isotropic simplification (most clearly on the macroscale and on longer timescales, with evidence from applications of neural field theory to experimental data going back decades, but also including the recent high-profile Pang results) can be a powerful approximation for brain dynamics, as well as drawing attention to settings in which this geometric approximation fails and where the contribution of long-range connections is dominant (most clearly on the microscale and on shorter timescales). This apparent paradox—where the geometric assumption is sometimes powerful but in other settings very weak—is at the center of our paper.

We fully agree with the reviewer’s point about spatial scale being a crucial nuance. We aimed to capture this in our framing of the study, e.g., in the opening paragraph of our Discussion:

*This work aims to address two lines of evidence regarding the functional role of fast-conducting non-local projections (FNPs) on cortical dynamics. One range of experiments demonstrate the critical role of specific, genetically inherited FNPs in shaping the intricate spatiotemporal dynamics of cortical activity, such as the clear non-local activations seen in voltage-sensitive dye (VSD) imaging of stimulus-evoked cortical responses in mouse [Refs], or long-range correlations between cortical populations mediated by structural connectivity [Refs]. These empirical results, across scales and species, demonstrate the important role of FNP networks in facilitating the complex, spatially distributed cortico-cortical communication thought to underpin the brain's remarkable cognitive functionality, despite their costs to develop, maintain and operate [Refs]. On the other hand, a second collection of results demonstrates the success of neural field models which, via a spatially homogeneous and isotropic connectivity rule assumption \textit{neglect} specific FNPs, but can nevertheless predict many aspects of cortical dynamics accurately, particularly the spontaneous fluctuations observed on the order-of-seconds timescales of fMRI [Refs]. This work sought to address the question: how can models of cortical dynamics that `smear out' the anatomical specificity of FNPs to a geometric average nevertheless generate such accurate predictions of cortical dynamics?*

Our results speak to this point by resolving the apparent paradox, arguing with the support of numerical experiments that the geometric approximation holds in the low-resolution limit (when averaging over long timescales), as in fMRI. We think that the appropriate level of balance and nuance on these points is contained in our presentation of the problem and its subtlety as well as our interpretation of the results in this context.

***Comment 3.4***

*3. I appreciate the abstractness of the model, but I worry that it doesn't make a strong connection to empirical, biological observation. I suppose this is expected from a "phenomenological model," but still seems like the connection could be made more explicit.*

**Response 3.4**

This paper aims to make general claims about models that include two key phenomenological mechanisms: (i) geometric spreading as travelling waves; and (ii) rapid non-local connectomic propagation. Accordingly, we developed and analyzed a phenomenological model that captures these two mechanisms as generally as possible, as they are shared by many specifically formulated models that may also contain additional mechanisms and thus differ in their precise formulation, parameter sets, etc. Additional discussion of this point is in the response to Reviewer 1 above: Response 1.1.

Despite the phenomenological formulation of our model, we note the connection to experiment via the physical units used in the model’s parameters, e.g., in terms of concrete lengthscales and timescales (in Table 1) that permit experimental testing of the model predictions.

While the benefits of our model’s phenomenological formulation for our purposes come at the expense of it being unable to describe spatiotemporal patterns in terms of concrete neurophysiological mechanisms, we note that this would be unreliable even for biophysically formulated models. Indeed, the reliability of inferring specific physiological mechanisms from measured macroscale brain dynamics on the longer timescales of fMRI using biophysically plausible models remains an open question and active area of development; cf. their outperformance by simpler phenomenological models in numerous such settings (cf. Response 1.1 above).

***Comment 3.5***

*Minor comments*

*1. P1, abstract: The authors state that axonal connections fundamentally shape function/dynamics. I agree, but it be worthwhile to note that at other scales, e.g. single neuron resolution, there is increased interest in extrasynaptic modes of communication, e.g. neuropeptide signaling networks, which do not depend on the connectome (Ripoll-Sanchez et al 2023, Neuron 10.1016/j.neuron.2023.09.043).*

**Response**:

Thank you for raising this interesting point; we have now incorporated it into the updated Discussion:

*A notable limitation of this work is that our modeling results, and their interpretation, rest on specific assumptions made in constructing the model.*

*[...]*

*The validity of the activity-preserving assumption that underlies the construction of our model (and the particular mathematical treatment of this assumption, cf. \cite{robinsonNearcriticalCorticothalamicEigenmodes2025}) could be interrogated further in future work.* ***Furthermore, emerging work suggests that neuronal communication can also take the form of extrasynaptic, `wireless' signalling through neuropeptides \cite{ripoll-sanchezNeuropeptidergicConnectomeElegans2023}, which is currently absent from this model.*** *Last, our model also assumes a system gain or excitability (model parameter $\nu_0$) that is constant over time, however it is known that the brain can operate at different levels of excitability over time, regulated by neuromodulatory systems of the subcortical brainstem and forebrain \cite{munnAscendingArousalSystem2021, orlandoNoradrenergicCholinergicSystems2023}.*

*[...]*

***Comment 3.6***

*2. P4: I don't understand the justification for preserving total activity of the model. Can the authors provide some justification why this is necessary or even a desired feature of the model? It strikes me as unrealistic.*

**Response 3.6**

We agree that this is an important assumption made in our model; different prior model formulations differ on this property. We made this choice to prevent dynamic instability when increasing the number of FNPs into the model. Our paper explains/justifies this choice in the *Methods*:

*A notable property of the non-local term $C\circ\phi$ is that, for any given input $f$, the total activity in the system integrated over space and time is preserved after the addition of any FNP to the structural connectivity. This property prevents non-oscillatory instabilities in the dynamics, which can occur when activity is regenerated from FNPs at a faster rate than activity globally dissipates \cite{qubbajNeuralFieldDynamics2007, jirsaNeuralFieldDynamics2009}, and therefore allows multiple FNPs to be added with arbitrary connectivity strengths. In alternative models, total activity is not preserved (e.g., the neural field model by Jirsa and colleagues \cite{qubbajNeuralFieldDynamics2007, jirsaNeuralFieldDynamics2009, jirsaSpatiotemporalPatternFormation2000}), such that the connectivity strengths of each FNP must be progressively reduced to maintain linear stability.*

And the limitations/implications of this property of our model are discussed in the Discussion:

*A notable limitation of this work is that our modeling results and their interpretation rest on specific assumptions made in constructing the model. One particular notable property of the model is its preservation of the total system activity over time (e.g., in `transporting' activity from one location to another, FNPs preserve total activity). This property stands in contrast to some other models, in which FNPs can propagate activity to multiple target locations and increase total activity, and nonlinearities in the local dynamics prevent dynamical instabilities arising from this increase \cite{decoRareLongrangeCortical2021}. The validity of the activity-preserving assumption that underlies the construction of our model (and the particular mathematical treatment of this assumption, cf. \cite{robinsonNearcriticalCorticothalamicEigenmodes2025}) could be interrogated further in future work.*

In addition to its convenience for building a model that cannot become unstable with the addition of new FNPs, its biological justification is treated in the neural field theory literature. Here, activity conversation arises from the model mechanism that every pulse at the synapse (variable phi) arises from a pulse that is generated at the soma (variable *Q*). Hence, the total number of synaptic pulses (sum phi) is equal to the total number of pulses (sum *Q*). This means that when an FNP is added then the number of pulses through geometric propagation must decrease by the number of pulses that is propagated via the FNP. We have now further elaborated on this point in the supporting information:

*Finally we aim to calibrate the effective gain terms $\tilde{G}_e(\mathbf{r}), \tilde{G}_w(\mathbf{r})$ in space, to satisfy the property that, for any given input $f(\mathbf{r}, t)$, the total activity in the system integrated over space and time is preserved after the addition of population $w$.*

***This preservation follows the approach through which neural field models are constructed, in which the total spacetime-integrated propagation of activity is preserved \cite{robinsonNearcriticalCorticothalamicEigenmodes2025}.*** *We let $\phi_0(\mathbf{r}, t)$ be the solution to the system in the absence of population $w$.*

[...]

***Comment 3.7***

*3. Fig. 2: The result is intuitive, but I wonder if it could have been any other outcome. If you add long-distance projections, you must disrupt the pure propagating wave, right? What is the alternative?*

**Response 3.7**

We agree with the reviewer’s intuition that fast long-distance projections must disrupt the geometric model, as shown in Figure 2. Our key contribution here is to capture the timescales over which the perturbation unfolds (and later how it depends on the properties of a targeted stimulation responses versus spontaneous dynamics), and in particular characterizing the result that on long timescales the perturbation resolves such that the dynamics approach that of the unperturbed dynamics. This implies that on these longer timescales, the dynamics are better-approximated by a geometric-average ‘smearing’ of the specificity of connectivity, as is commonly done in neural field theories, but on rapid timescales the contribution of rapid long-distance projections leads to highly specific and non-local dynamical patterns (as seen clearly in e.g., whisker stimulation experiments) and cannot be neglected or averaged over.

We also note a connection between the reviewer’s comment and how the propagating wave can be “disrupted” via destructive interference, a possibility that is observed experimentally in some trials in visual cortex (Xu et al. Neuron 2007). This requires a nonlinear interaction, which although cannot be captured by our linear model, is an interesting direction to be potentially explored in future modeling work. We have updated the Discussion to now reference this potential:

*[…]*

*For example, simulations with the neural field model by Steyn-Ross et al. \cite{steyn-rossInteractingTuringHopfInstabilities2013}, which generalizes the model by Jirsa et al. by introducing realistic nonlinear mechanisms, suggest that introducing a bidirectional FNP can induce seizure-like bursting activity when electrical gap-junction synapses between inhibitory neurons are introduced, but otherwise minimally affects the phase coherence statistics of spontaneous dynamics.* ***Future models could also include nonlinear mechanisms to capture more realistic interactions of interfering traveling waves, such as compression and reflection in visual cortex \cite{xuCompressionReflectionVisually2007}.***

***Comment 3.8***

*4. The authors make claims about the universality of their model (great for studying fast dynamics and slower dynamics). How are these equated? VSD and fMRI have very different intrinsic timescales.*

**Response 3.8**

Our model simulates fast-timescale dynamics on which specific fast non-local projections strongly shape the resulting neural dynamics. These fast dynamics are experimentally accessible via modalities with high temporal resolution (and ideally also sufficiently high spatial resolution) such as VSD. When we can only access slower dynamics experimentally (as in fMRI), our model predicts that such nonlocal dynamics are ‘washed out’ and on such timescales can be better approximated by geometric models. So by numerically simulating predicted patterns of neural dynamics at high temporal resolution, our model is able to differentiate how different dynamics may play out on different timescales. This ability is core to the claims made in our paper about why different dynamical mechanisms may play different roles on different such timescales.
