# Supplementary material for: Modeling the influences of non-local connectomic projections on geometrically constrained cortical dynamics
Source: PLoS Comput Biol. 2026 Aug 26;22(8):e1014673. doi: 10.1371/journal.pcbi.1014673 (PMC13552967; doi:10.1371/journal.pcbi.1014673)
Supplement: Peer review history

# Author comment: Author response to Decision Letter 0

**DOI:** 10.1371/journal.pcbi.1014673.r001

20 Mar 2026

Attachment Submitted filename: renamed_7c58a.docx

# Peer review: Decision Letter 0

**DOI:** 10.1371/journal.pcbi.1014673.r002

**Authors:** Thomas Serre, Roman Bauer

17 May 2026

PCOMPBIOL-D-26-00639

Modeling the influences of non-local connectomic projections on geometrically constrained cortical dynamics

PLOS Computational Biology

Dear Dr. Fulcher,

Thank you for submitting your manuscript to PLOS Computational Biology. After careful consideration, we feel that it has merit but does not fully meet PLOS Computational Biology's publication criteria as it currently stands. Therefore, we invite you to submit a revised version of the manuscript that addresses the points raised during the review process.

Please submit your revised manuscript by Jul 17 2026 11:59PM. If you will need more time than this to complete your revisions, please reply to this message or contact the journal office at ploscompbiol@plos.org. When you're ready to submit your revision, log on to https://www.editorialmanager.com/pcompbiol/ and select the 'Submissions Needing Revision' folder to locate your manuscript file.

Please include the following items when submitting your revised manuscript:

* A letter that responds to each point raised by the editor and reviewer(s). You should upload this letter as a separate file labeled 'Response to Reviewers'. This file does not need to include responses to formatting updates and technical items listed in the 'Journal Requirements' section below.

* A marked-up copy of your manuscript that highlights changes made to the original version. You should upload this as a separate file labeled 'Revised Manuscript with Track Changes'.

* An unmarked version of your revised paper without tracked changes. You should upload this as a separate file labeled 'Manuscript'.

If you would like to make changes to your financial disclosure, competing interests statement, or data availability statement, please make these updates within the submission form at the time of resubmission. Guidelines for resubmitting your figure files are available below the reviewer comments at the end of this letter.

As the corresponding author, your ORCID iD is verified in the submission system and will appear in the published article. PLOS supports the use of ORCID, and we encourage all coauthors to register for an ORCID iD and use it as well. Please encourage your coauthors to verify their ORCID iD within the submission system before final acceptance, as unverified ORCID iDs will not appear in the published article. Only the individual author can complete the verification step; PLOS staff cannot verify ORCID iDs on behalf of authors.

We look forward to receiving your revised manuscript.

Kind regards,

Roman Bauer

Academic Editor

PLOS Computational Biology

Thomas Serre

Section Editor

PLOS Computational Biology

**Additional Editor Comments :**

Please address the reviewers' comments. Once you've submitted the revised manuscript, it most likely does not need to be sent out for review again, i.e., we should be able to confirm that the comments are sufficiently addressed without the reviewers.

**Journal Requirements:**

1) Please ensure that the CRediT author contributions listed for every co-author are completed accurately and in full.

At this stage, the following Authors/Authors require contributions: Rishi Maran, Eli Muller, and Ben D. Fulcher. Please ensure that the full contributions of each author are acknowledged in the "Add/Edit/Remove Authors" section of our submission form.

The list of CRediT author contributions may be found here: https://journals.plos.org/ploscompbiol/s/authorship#loc-author-contributions

2) We ask that a manuscript source file is provided at Revision. Please upload your manuscript file as a .doc, .docx, .rtf or .tex. If you are providing a .tex file, please upload it under the item type u2018LaTeX Source Fileu2019 and leave your .pdf version as the item type u2018Manuscriptu2019.

3) Please upload all main figures as separate Figure files in .tif or .eps format. For more information about how to convert and format your figure files please see our guidelines:

https://journals.plos.org/ploscompbiol/s/figures

4) We notice that your supplementary Figures, and information are included in the manuscript file. Please remove them and upload them with the file type 'Supporting Information'. Please ensure that each Supporting Information file has a legend listed in the manuscript after the references list.

5) Please amend your detailed Financial Disclosure statement. This is published with the article. It must therefore be completed in full sentences and contain the exact wording you wish to be published.

1) State what role the funders took in the study. If the funders had no role in your study, please state: "The funders had no role in study design, data collection and analysis, decision to publish, or preparation of the manuscript.".

Note: If the reviewer comments include a recommendation to cite specific previously published works, please review and evaluate these publications to determine whether they are relevant and should be cited. There is no requirement to cite these works unless the editor has indicated otherwise.

**Reviewers' comments:**

Reviewer's Responses to Questions

**Comments to the Authors:**

**Please note that one review is uploaded as an attachment.**

Reviewer #1: The manuscript presents a clear and interesting theoretical contribution to the ongoing debate on the respective roles of cortical geometry and long-range connectivity in shaping macroscale brain dynamics. The authors introduce a hybrid neural field framework that combines geometrically constrained propagation on a continuous cortical sheet with discrete fast non-local projections, and they use this model to study how the contribution of these two mechanisms depends on timescale and input conditions. Overall, I enjoyed reading the manuscript, wich is well written, mathematically clear, and conceptually relevant. In particular, the main result — that fast non-local projections strongly affect rapid stimulus-evoked dynamics but have a much weaker influence on slower spontaneous fluctuations — is compelling and provides a useful reconciliation between geometry-based accounts and connectome-based accounts of resting-state activities. I only have a few minor suggestions, as follows.

First, I think the manuscript would benefit from a slightly more restrained statement of novelty. The important contribution, in my view, is not the coexistence of local geometric propagation and non-local projections within one framework, but the explicit demonstration that their relative importance depends strongly on temporal regime and input structure (even if some of the assumptions might be irrealistic at this stage). Clearly framing the advancement in those terms would make the paper more precise and, in fact, stronger in my view. This is important because the paper proposes a framework that addresses a tension currently present in the literature.

Also, one potential improvement could come if the authors would make the connection to empirical neuroscience a little more. Firstly, I would suggest to consider this manuscript (The structural connectome constrains fast brain dynamics), based on empirical data, as it is directly relevant to the manuscript's central clain. Also, the paper could more explicitly highlight which experimental settings are most relevant to the model’s predictions. In its current form, the manuscript already motivates the problem through fast evoked activity, resting-state fMRI, and related macroscale recordings, so this step would be quite natural.

In summary, this is a strong and thoughtful computational paper that addresses an important question with appropriate theoretical tools. I compliment the authors. The results are interesting, the manuscript is generally clear, and the main conclusions are worthy of publication in my opinion.

Reviewer #2: I reviewed the manuscript originally submitted to PLoS Biology. All reviewers concerned that the claims of biology relevance were not sufficiently supported as the modeling was based on simplifying assumptions. In this revised version submitted now to PLoS Computational Biology, the authors stressed that the model aimed to present two key phenomenological mechanisms: (i) geometric spreading as travelling waves; and (ii) rapid non-local connectomic propagation.. The authors expected similar qualitative behavior from a large class of specifically formulated models that contain the two high-level mechanisms that the paper (and phenomenological model) targets. While some properties of simulated dynamics of more complex models would better capture some complexities found in real data, the modeling results would be less clearly generalizable. Limitations of the models are more adequately discussed

Following the same logic, however, I would like to point that the emphasized novel finding that "FNPs shape the model dynamics of rapid, stimulus-evoked responses on fine millisecond timescales (⪅ 30 ms), but contribute relatively little to slower, spontaneous fluctuations over longer timescales (>30 ms)", may not be really generalizable, especially the quantitative (single) threshold of 30 ms, as this could be the result of the “specific” over-simplifying assumptions of the model (passive diffusion rather than the excitability property of the neural field, and simplified topology of FNP when compared to empirical connectomes). I would suggest that the authors make this point clearer already in Abstract, Introduction, not just in Discussion.

Apart from this point, in my view, the revised version is satisfactory for PLoS CB.

Reviewer #3: Review is updated as attachment.

**********

**Have the authors made all data and (if applicable) computational code underlying the findings in their manuscript fully available?**

The PLOS Data policy requires authors to make all data and code underlying the findings described in their manuscript fully available without restriction, with rare exception (please refer to the Data Availability Statement in the manuscript PDF file). The data and code should be provided as part of the manuscript or its supporting information, or deposited to a public repository. For example, in addition to summary statistics, the data points behind means, medians and variance measures should be available. If there are restrictions on publicly sharing data or code —e.g. participant privacy or use of data from a third party—those must be specified.

Reviewer #1: Yes

Reviewer #2: Yes

Reviewer #3: Yes

**********

PLOS authors have the option to publish the peer review history of their article (what does this mean?). If published, this will include your full peer review and any attached files.

If you choose “no”, your identity will remain anonymous but your review may still be made public.

**Do you want your identity to be public for this peer review?**  For information about this choice, including consent withdrawal, please see our Privacy Policy.

Reviewer #1: No

Reviewer #2: No

Reviewer #3: No

[NOTE: If reviewer comments were submitted as an attachment file, they will be attached to this email and accessible via the submission site. Please log into your account, locate the manuscript record, and check for the action link "View Attachments". If this link does not appear, there are no attachment files.]

**Figure resubmission:**

**Reproducibility:**

To enhance the reproducibility of your results, we recommend that authors of applicable studies deposit laboratory protocols in protocols.io, where a protocol can be assigned its own identifier (DOI) such that it can be cited independently in the future. Additionally, PLOS ONE offers an option to publish peer-reviewed clinical study protocols. Read more information on sharing protocols at https://plos.org/protocols?utm_medium=editorial-email&utm_source=authorletters&utm_campaign=protocols

Attachment Submitted filename: PLOS_CB_REVIEW.pdf

# Author comment: Author response to Decision Letter 1

**DOI:** 10.1371/journal.pcbi.1014673.r003

12 Jun 2026

Attachment Submitted filename: R2R - PLOSCB_June.pdf

# Editor report: Decision Letter 1

**DOI:** 10.1371/journal.pcbi.1014673.r004

**Authors:** Thomas Serre, Roman Bauer

1 Jul 2026

PCOMPBIOL-D-26-00639R1

Modeling the influences of non-local connectomic projections on geometrically constrained cortical dynamics

PLOS Computational Biology

Dear Dr. Fulcher,

Thank you for submitting your manuscript to PLOS Computational Biology. After careful consideration, we feel that it has merit but does not fully meet PLOS Computational Biology's publication criteria as it currently stands. Therefore, we invite you to submit a revised version of the manuscript that addresses the points raised during the review process.

Please submit your revised manuscript by Aug 31 2026 11:59PM. If you will need more time than this to complete your revisions, please reply to this message or contact the journal office at ploscompbiol@plos.org. When you're ready to submit your revision, log on to https://www.editorialmanager.com/pcompbiol/ and select the 'Submissions Needing Revision' folder to locate your manuscript file.

Please include the following items when submitting your revised manuscript:

* A letter that responds to each point raised by the editor and reviewer(s). You should upload this letter as a separate file labeled 'Response to Reviewers'. This file does not need to include responses to formatting updates and technical items listed in the 'Journal Requirements' section below.

* A marked-up copy of your manuscript that highlights changes made to the original version. You should upload this as a separate file labeled 'Revised Manuscript with Track Changes'.

* An unmarked version of your revised paper without tracked changes. You should upload this as a separate file labeled 'Manuscript'.

If you would like to make changes to your financial disclosure, competing interests statement, or data availability statement, please make these updates within the submission form at the time of resubmission. Guidelines for resubmitting your figure files are available below the reviewer comments at the end of this letter.

As the corresponding author, your ORCID iD is verified in the submission system and will appear in the published article. PLOS supports the use of ORCID, and we encourage all coauthors to register for an ORCID iD and use it as well. Please encourage your coauthors to verify their ORCID iD within the submission system before final acceptance, as unverified ORCID iDs will not appear in the published article. Only the individual author can complete the verification step; PLOS staff cannot verify ORCID iDs on behalf of authors.

We look forward to receiving your revised manuscript.

Kind regards,

Roman Bauer

Academic Editor

PLOS Computational Biology

Thomas Serre

Section Editor

PLOS Computational Biology

**Additional Editor Comments (if provided):**

**Journal Requirements:**

If the reviewer comments include a recommendation to cite specific previously published works, please review and evaluate these publications to determine whether they are relevant and should be cited. There is no requirement to cite these works unless the editor has indicated otherwise.

1) Please upload all main figures as separate Figure files in .tif or .eps format. For more information about how to convert and format your figure files please see our guidelines:

https://journals.plos.org/ploscompbiol/s/figures

2) Your current Financial Disclosure states, “B.D.F. received support from the Australian Research Council (FT240100418, DP240101295). E.J.M. acknowledges support from the Australian Research Council (DE250100540). The funders had no role in study design, data collection and analysis, decision to publish, or preparation of the manuscript”. However, in your funding information on the submission form, "order of grants" mismatches. Please indicate by return email the full and correct funding information for your study and confirm the order in which funding contributions should appear. Please be sure to indicate whether the funders played any role in the study design, data collection and analysis, decision to publish, or preparation of the manuscript.

**Reviewers' comments:**

[NOTE: If reviewer comments were submitted as an attachment file, they will be attached to this email and accessible via the submission site. Please log into your account, locate the manuscript record, and check for the action link "View Attachments". If this link does not appear, there are no attachment files.]

**Figure resubmission:**

**Reproducibility:**

To enhance the reproducibility of your results, we recommend that authors of applicable studies deposit laboratory protocols in protocols.io, where a protocol can be assigned its own identifier (DOI) such that it can be cited independently in the future. Additionally, PLOS ONE offers an option to publish peer-reviewed clinical study protocols. Read more information on sharing protocols at https://plos.org/protocols?utm_medium=editorial-email&utm_source=authorletters&utm_campaign=protocols

# Author comment: Author response to Decision Letter 2

**DOI:** 10.1371/journal.pcbi.1014673.r005

13 Jul 2026

Attachment Submitted filename: R2R_-_PLOSCB_June_auresp_2.pdf

# Peer review: Decision Letter 2

**DOI:** 10.1371/journal.pcbi.1014673.r006

**Authors:** Thomas Serre

5 Aug 2026

Dear Dr. Fulcher,

We are pleased to inform you that your manuscript 'Modeling the influences of non-local connectomic projections on geometrically constrained cortical dynamics' has been provisionally accepted for publication in PLOS Computational Biology.

Before your manuscript can be formally accepted you will need to complete some formatting changes, which you will receive in a follow up email. A member of our team will be in touch with a set of requests.

Please note that your manuscript will not be scheduled for publication until you have made the required changes, so a swift response is appreciated.

IMPORTANT: The editorial review process is now complete. PLOS will only permit corrections to spelling, formatting or significant scientific errors from this point onwards. Requests for major changes, or any which affect the scientific understanding of your work, will cause delays to the publication date of your manuscript.

Should you, your institution's press office or the journal office choose to press release your paper, you will automatically be opted out of early publication. We ask that you notify us now if you or your institution is planning to press release the article. All press must be co-ordinated with PLOS.

Thank you again for supporting Open Access publishing; we are looking forward to publishing your work in PLOS Computational Biology.

Best regards,

Thomas Serre

Section Editor

PLOS Computational Biology

Thomas Serre

Section Editor

PLOS Computational Biology

***********************************************************

Reviewer's Responses to Questions

**Comments to the Authors:**

**Please note here if the review is uploaded as an attachment.**

Reviewer #1: I thank the authors for addressing my concerns. I have no further remarks.

Reviewer #2: The authors have made revision to address my concerns. It is acceptable for publication in PLoS CB.

Reviewer #3: All my comments and suggestions have been addressed, including additional checks on spatio-temporal patterns, and a clear explanation on why these do not reveal further insights. I recommend publication.

**********

**Have the authors made all data and (if applicable) computational code underlying the findings in their manuscript fully available?**

The PLOS Data policy requires authors to make all data and code underlying the findings described in their manuscript fully available without restriction, with rare exception (please refer to the Data Availability Statement in the manuscript PDF file). The data and code should be provided as part of the manuscript or its supporting information, or deposited to a public repository. For example, in addition to summary statistics, the data points behind means, medians and variance measures should be available. If there are restrictions on publicly sharing data or code —e.g. participant privacy or use of data from a third party—those must be specified.

Reviewer #1: Yes

Reviewer #2: Yes

Reviewer #3: Yes

**********

PLOS authors have the option to publish the peer review history of their article (what does this mean?). If published, this will include your full peer review and any attached files.

If you choose “no”, your identity will remain anonymous but your review may still be made public.

**Do you want your identity to be public for this peer review?**  For information about this choice, including consent withdrawal, please see our Privacy Policy.

Reviewer #1: No

Reviewer #2: No

Reviewer #3: No

# Editor report: Acceptance letter

**DOI:** 10.1371/journal.pcbi.1014673.r007

**Authors:** Thomas Serre

PCOMPBIOL-D-26-00639R2

Modeling the influences of non-local connectomic projections on geometrically constrained cortical dynamics

Dear Dr Fulcher,

I am pleased to inform you that your manuscript has been formally accepted for publication in PLOS Computational Biology. Your manuscript is now with our production department and you will be notified of the publication date in due course.

The corresponding author will soon be receiving a typeset proof for review, to ensure errors have not been introduced during production. Please review the PDF proof of your manuscript carefully, as this is the last chance to correct any errors. Please note that major changes, or those which affect the scientific understanding of the work, will likely cause delays to the publication date of your manuscript.

Soon after your final files are uploaded, unless you have opted out, the early version of your manuscript will be published online. The date of the early version will be your article's publication date. The final article will be published to the same URL, and all versions of the paper will be accessible to readers.

For Research, Software, and Methods articles, you will receive an invoice from PLOS for your publication fee after your manuscript has reached the completed accept phase. If you receive an email requesting payment before acceptance or for any other service, this may be a phishing scheme. Learn how to identify phishing emails and protect your accounts at https://explore.plos.org/phishing.

Thank you again for supporting PLOS Computational Biology and open-access publishing. We are looking forward to publishing your work!

With kind regards,

Sharmila Kamatchi

PLOS Computational Biology | Carlyle House, Carlyle Road, Cambridge CB4 3DN | United Kingdom ploscompbiol@plos.org | Phone +44 (0) 1223-442824 | ploscompbiol.org | @PLOSCompBiol
